# New Pyrazolopyrimidine Inhibitors of Protein Kinase D as Potent Anticancer Agents for Prostate Cancer Cells

**DOI:** 10.1371/journal.pone.0075601

**Published:** 2013-09-23

**Authors:** Manuj Tandon, James Johnson, Zhihong Li, Shuping Xu, Peter Wipf, Qiming Jane Wang

**Affiliations:** 1 Department of Pharmacology and Chemical Biology, University of Pittsburgh, Pittsburgh, Pennsylvania, United States of America; 2 Department of Chemistry and Center for Chemical Methodologies and Library Development, University of Pittsburgh, Pittsburgh, Pennsylvania, United States of America; Tohoku University, Japan

## Abstract

The emergence of protein kinase D (PKD) as a potential therapeutic target for several diseases including cancer has triggered the search for potent, selective, and cell-permeable small molecule inhibitors. In this study, we describe the identification, *in vitro* characterization, structure-activity analysis, and biological evaluation of a novel PKD inhibitory scaffold exemplified by 1-naphthyl PP1 (1-NA-PP1). 1-NA-PP1 and IKK-16 were identified as pan-PKD inhibitors in a small-scale targeted kinase inhibitor library assay. Both screening hits inhibited PKD isoforms at about 100 nM and were ATP-competitive inhibitors. Analysis of several related kinases indicated that 1-NA-PP1 was highly selective for PKD as compared to IKK-16. SAR analysis showed that 1-NA-PP1 was considerably more potent and showed distinct substituent effects at the pyrazolopyrimidine core. 1-NA-PP1 was cell-active, and potently blocked prostate cancer cell proliferation by inducing G2/M arrest. It also potently blocked the migration and invasion of prostate cancer cells, demonstrating promising anticancer activities on multiple fronts. Overexpression of PKD1 or PKD3 almost completely reversed the growth arrest and the inhibition of tumor cell invasion caused by 1-NA-PP1, indicating that its anti-proliferative and anti-invasive activities were mediated through the inhibition of PKD. Interestingly, a 12-fold increase in sensitivity to 1-NA-PP1 could be achieved by engineering a gatekeeper mutation in the active site of PKD1, suggesting that 1-NA-PP1 could be paired with the analog-sensitive PKD1^M659G^ for dissecting PKD-specific functions and signaling pathways in various biological systems.

## Introduction

Following the discovery of Gleevec, it was widely demonstrated that highly potent and specific small molecule inhibitors of kinases exhibit remarkable clinical efficacy and reduced toxicity [1]. Protein kinases are key regulators of signal transduction pathways and therefore attractive therapeutic targets for many diseases. The serine/threonine protein kinase D (PKD) family forms a distinct group of calcium/calmodulin-dependent protein kinases (CAMK) [2], [3]. The three known isoforms of PKD (PKD1, PKD2 and PKD3) play important roles in several fundamental cellular processes, including cell proliferation, survival, migration, gene regulation, protein trafficking, and immune response [4], [5]. In particular, PKD1 has been implicated in many aspects of tumor development, such as tumor growth, metastasis, and angiogenesis [4]. Aberrant PKD activity and expression have been reported in various tumor cell lines and tumor tissues from the pancreas [5], skin [6], [7] and prostate [8], [9]. PKD mediates major signaling pathways that are vital to cancer development, including the VEGF and MEK/ERK signaling pathways [4], supporting an active role of PKD in tumor-associated biological processes in diverse cancer types [5], [7], [9]–[12].

PKD is a key signaling component of the diacylglycerol (DAG) signaling network. It is a primary target of DAG and a downstream effector of protein kinase C (PKC). There are several conserved structural motifs in PKD, such as a C1 domain that binds DAG and modulates PKD localization, and a PH domain that exerts an autoinhibitory function on the kinase domain. In intact cells, PKD is directly phosphorylated by DAG-responsive PKCs on the two conserved serine residues in the activation loop of the catalytic domain, which leads to its activation. PKD often exhibits sustained activity upon activation which is mainly maintained through autophosphorylation [5].

In the past several years, significant progress has been made in the development of potent and specific small molecule PKD inhibitors. CID755673 and analogs [13], [14], 2,6-naphthyridine and bipyridyl inhibitors and their analogs [15]–[17], 3,5-diarylazoles [18], CRT0066101 [19], and CRT5 [20] demonstrated targeted inhibition of PKD *in vitro* and in intact cells. Particularly, CRT0066101 was found to potently block the growth of pancreatic tumor xenografts *in vivo* in mice [19]. Despite these significant advances, no PKD-subtype specific inhibitor is available, and PKD inhibitors have yet to progress to the clinic. In part, the absence of clinical candidates can be attributed to the limited selectivity, *in vivo* stability and general toxicity issues with the current set of known inhibitors. Therefore, it is imperative to continue the search for novel PKD inhibitory chemotypes that demonstrate attractive target selectivity, improved pharmacokinetic profiles, and greater *in vivo* efficacy.

We have previously identified both ATP-competitive and -noncompetitive PKD inhibitors that are distinct in structure to other reported inhibitors [13], [14], [21]–[23]. These hits were identified in a HTS campaign using large, unbiased small molecule libraries. Subsequently, medicinal chemistry strategies were used to optimize the activity, selectivity, and physicochemical properties of a lead structure, CID755673, resulting in a series of analogs that showed enhanced target inhibition *in vitro* and in cells, and improved metabolic profiles [13], [14], [21]–[24]. Herein, we describe the identification and evaluation of a novel PKD inhibitory chemotype based on 1-naphthyl PP1 (1-NA-PP1), a pyrazolopyrimidine that was originally designed for the analog-sensitive mutant kinase of src [25]. This inhibitor was identified in a small, targeted library of diverse kinase inhibitors. 1–NA-PP1 exhibited excellent selectivity towards PKD with little or no inhibitory activity for two related kinases, CAMK or PKC. It potently inhibited the proliferation, migration and invasion of prostate cancer cells. A subsequent SAR analysis revealed important structural determinants for this lead compound and positions 1-NA-PP1 as a new and distinct PKD inhibitor chemotype with the potential to yield development candidates for *in vitro* and *in vivo* applications.

## Results

### Identification of novel PKD inhibitory scaffolds from a targeted kinase inhibitor library

Eighty chemically diverse kinase inhibitors were selected from a Tocris Biosciences small molecule collection. The *in vitro* PKD1 inhibitory activity of these compounds was evaluated based on their ability to inhibit the recombinant PKD1 protein at 1 µM concentration in a radiometric PKD kinase assay. The percent PKD1 inhibition was calculated as the percent inhibition of the total PKD1 kinase activity in the absence of inhibitors (DMSO). Sixteen compounds were identified as primary hits (≥50% inhibition of total PKD1 kinase activity) in the radiometric PKD1 assay (**Table 1**). Among these hits, 1-NA-PP1, a mutant src kinase inhibitor, and IKK-16, an IκB kinase inhibitor, suppressed 77% and 67% of PKD1 activity at 1 µM, respectively, and were selected for further characterization based on their potency and distinct structural features.

**Table 1:** Primary hits identified in a PKD1 inhibitor screen of a targeted library.

*Compound Name*	*Known target*	*% PKD1 Inhibition*
Fasudil hydrochloride	ROCK	41%
SP 600125	JNK	74%
Ro 31-8220 Mesylate	PKC, MAPK, ERK2	72%
Arcyriaflavin A	Cyclin D1/CAMK II	75%
**IKK-16**	**IκB kinase**	**67%**
SB 218078	Check point Kinase I	85%
PD 407824	Check point Kinase I	57%
D 4476	casein kinase 1	45%
EO 1428	p38 MAPK	68%
H 89 Dihydrochloride	PKA	66%
Iressa	EGFR	45%
SU 5416	VEGFR	45%
**1-NA-PP1**	**Src mutant**	**77%**
Dorsomorphin dihydrochloride	AMPK	50%
BIO	GSK-3	47%
SD 208	TGF-βRI	74%
kb-NB142-70	PKD	88%

A targeted protein kinase inhibitor library of 80 compounds was screened for PKD1 inhibitory activity at 1 µM using an *in vitro* radiometric PKD1 kinase assay. Sixteen compounds were selected as primary hits based on their ability to inhibit PKD1 at or above 50% at 1 µM. The % PKD1 inhibition referred to the percent inhibition of the total kinase activity measured in the absence of inhibitors (DMSO). Kb-NB142-70, a previously validated PKD inhibitor, was used as a positive control. Experiments were performed with triplicate determinations at 1 µM for each compound.

### 1-NA-PP1 and IKK-16 are novel pan-PKD inhibitors

**Figure 1:**
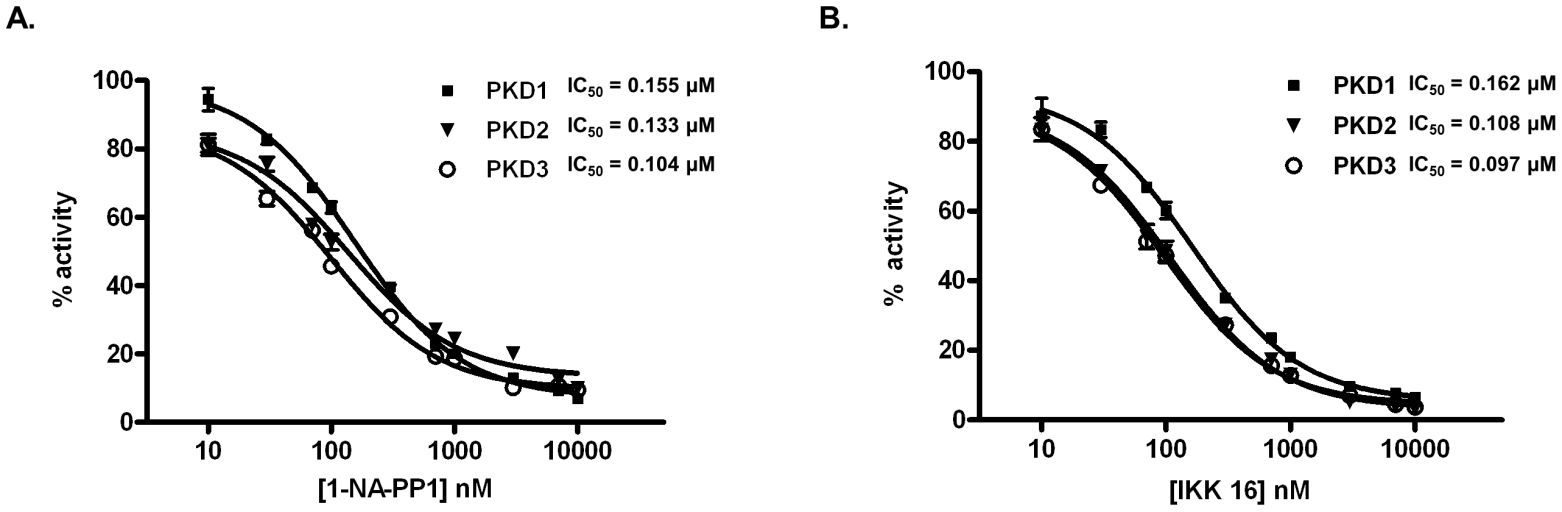
Inhibition of PKD isoforms by 1-NA-PP1 and IKK-16. Inhibition of recombinant human PKD1, 2 and 3 was assayed in the presence of 10 different concentrations of 1-NA-PP1 (**A**) and IKK-16 (**B**) by an *in vitro* radiometric PKD kinase assay. The IC_50_ values were calculated as the mean ±SEM of at least three independent experiments with triplicate determinations at each concentration of drug in each experiment. The data were plotted as a function of drug concentration and a representative graph is shown.

The *in vitro* IC_50_ of 1-NA-PP1 and IKK-16 was determined in a 10-point concentration curve using a radiometric PKD kinase assay [13]. Recombinant human PKD1, 2, or 3 proteins were incubated in a mixture containing a peptide substrate derived from a PKD substrate, HDAC-5, and 10 different concentrations of the two compounds. As shown in **Fig. 1A**, 1-NA-PP1 and IKK 16 inhibited all three isoforms of PKD with nearly equal potency. 1-NA-PP1 inhibited PKD1, 2, and 3 with an IC_50_ of 154.6±21.8 nM (n = 3), 133.4+/−3.6 nM (n = 3), 109.4+/−6.8 nM (n = 3), respectively, while IKK-16 similarly inhibited the PKD isoforms with an IC_50_ of 153.9+/−7.7 nM (n = 2) for PKD1, 115.0+/−7.1 nM (n = 2) for PKD2, and 99.7+/−3.0 nM (n = 2) for PKD3. These results indicate that both 1-NA-PP1 and IKK 16 are potent pan-PKD inhibitors.

### 1-NA-PP1 is an ATP-competitive inhibitor with high selectivity for PKD over closely related kinases

**Figure 2:**
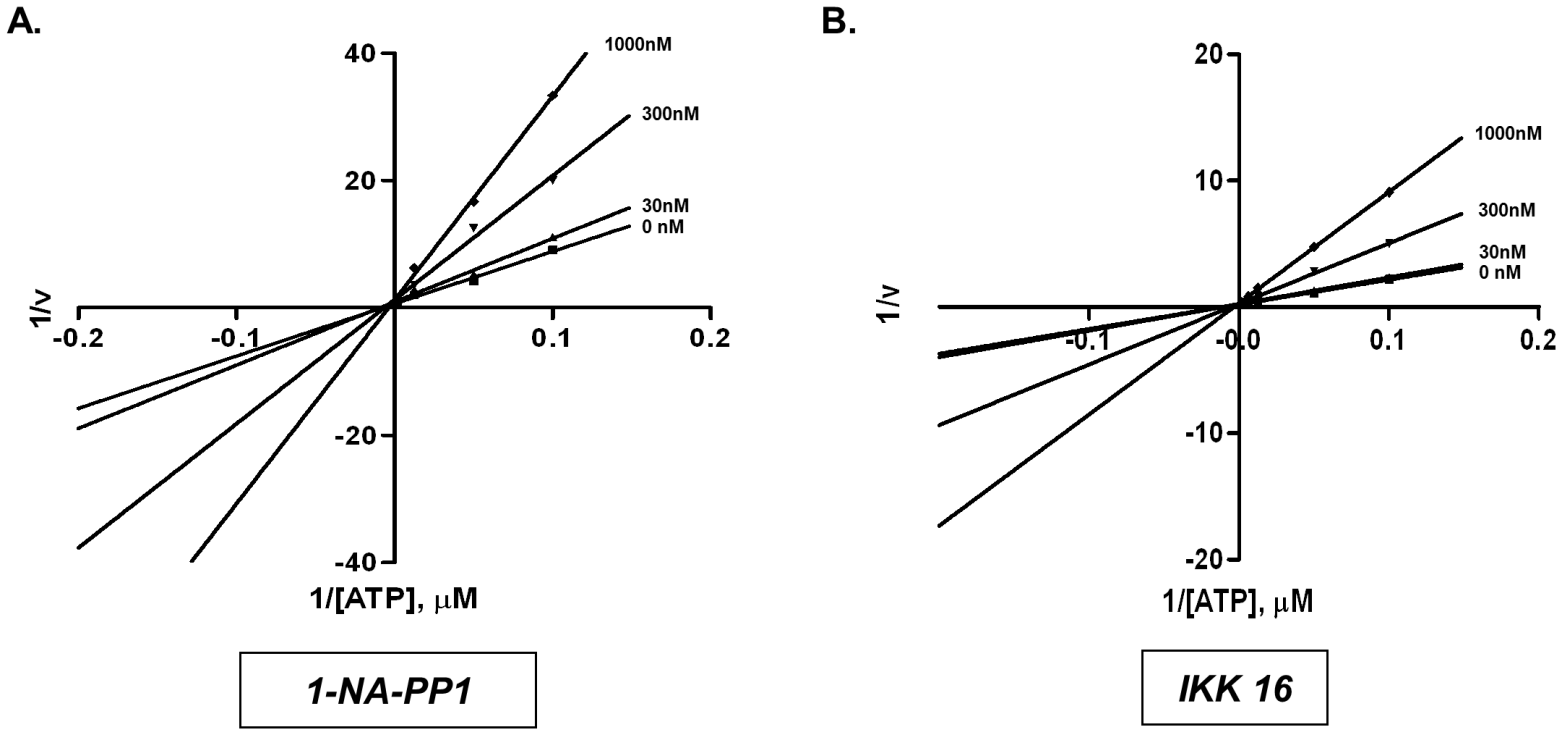
IKK-16 and 1-NA-PP1 were ATP-competitive inhibitors of PKD. PKD1 kinase activity was measured as a function of increasing concentrations of ATP in the presence of varying concentrations of 1-NA-PP1 (**A**) and IKK-16 (**B**). Lineweaver-Burke plots of the data are shown. Data presented were representative of three independent experiments.

To gain a better understanding of the mode of action for 1-NA-PP1 and IKK-16, we examined the effects of increasing concentrations of ATP on PKD1 inhibition. Lineweaver-Burk plots were generated by plotting the reciprocal of reaction velocities (1/v) against the reciprocal of ATP concentrations (1/[ATP]) at different compound concentrations. The points were fitted by linear regression. As shown **Fig. 2A–B**, all lines converged on the Y-axis, indicating that both 1-NA-PP1 and IKK-16 were ATP-competitive inhibitors.

**Figure 3:**
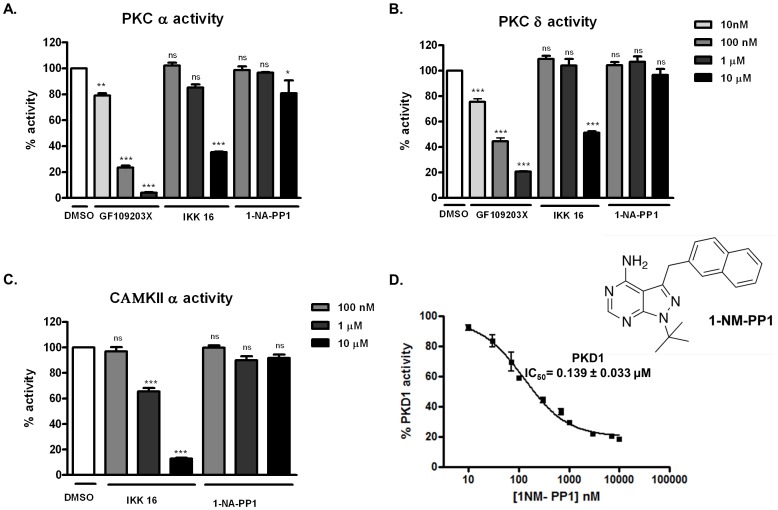
1-NA-PP1 did not inhibit PKC and CAMK. Inhibition of PKCα (**A**) or PKCδ (**B**) was determined at 10 nM, 100 nM, 1 µM, and 10 µM. As controls, the PKC inhibitor GF109203X potently inhibited PKCα and PKCδ activity. Data are the mean ±SEM of two independent experiments. **C.** Inhibition of CAMKIIα was measured by the radiometric CAMK kinase assay. The experiment was repeated twice and a representative graph is shown. Statistical significance was determined using the unpaired t-test. ns, not statistically significant; *, *p*<0.05; **, *p*<0.01; ***, *p*<0.001.

Next, we determined the specificity of 1-NA-PP1 and IKK-16 for PKD by examining their activity for several functionally or structurally related kinases, including PKCα, PKCδ and CAMKIIα. No significant inhibitory activities for PKC isoforms were detected at 0.1, 1 and 10 µM concentrations of 1-NA-PP1, in contrast to IKK-16 which showed a concentration-dependent inhibition for both PKCα and PKCδ and >50% inhibition at 10 µM concentration (**Fig. 3A–B**). The potent PKC inhibitor GF109203X was tested as a positive control and it potently and concentration-dependently inhibited PKCα and PKCδ. PKD belongs to a subgroup of the CAMK family and kinases in both families share high sequence homology. This prompted us to evaluate the inhibition of CAMKIIα by these compounds. As illustrated in **Fig. 3C**, 1-NA-PP1 showed little activity on CAMKIIα up to 10 µM, while IKK-16 caused concentration-dependent inhibition of the enzyme and almost completely abrogated its activity at 10 µM. Taken together, these data indicate that 1-NA-PP1 is a highly specific inhibitor for PKD relative to other closely related kinases including PKCs and CAMKs, while IKK-16 is likely a promiscuous kinase inhibitor.

Further insights on the specificity of 1-NA-PP1 could be gained by evaluating the kinome scan data on 1-naphthylmethyl PP1 (1-NM-PP1), where 1-NM-PP1, along with 178 known kinase inhibitors, was profiled against a panel of 300 recombinant human protein kinases at a single concentration [26]. Like 1-NA-PP1, 1-NM-PP1 is also a C3-derivatized PP1 analog that differs from 1-NA-PP1 by only one methyl group linking pyrazole and naphthyl rings. Our data showed that 1-NM-PP1 inhibited PKD1 with an IC_50_ of 138.7±33.2 nM (n = 3) (**Fig. 3D**), which was equivalent to that of 1-NA-PP1 (154.6 nM). Their inhibitory activities for majority of the wild-type and mutated Src family kinases are also comparable [27], indicating that the two inhibitors are not only similar in structure but also in biological activity. The specificity data for 1-NM-PP1 were extracted at a cut-off of <50% residual kinase activity using the Kinase Inhibitor Resource (KIR) online tool (http://kir.fccc.edu/) as described (**Table S1**) [26]. The data confirmed the inhibition of PKD isoform by this compound. Although additional targets of 1-NM-PP1 were identified, this inhibitor exhibited a relative high Gini score of 0.67 (a selectivity score for kinases and kinase inhibitors), indicating higher selectivity. Meanwhile, no PKC isoforms were significantly inhibited by this inhibitor (>90% residual kinase activity for all PKC isoforms). Based on this and the above results, we choose to focus solely on 1-NA-PP1 in our subsequent studies.

### Synthesis and SAR analysis of 1-NA-PP1 analogs

**Figure 4:**
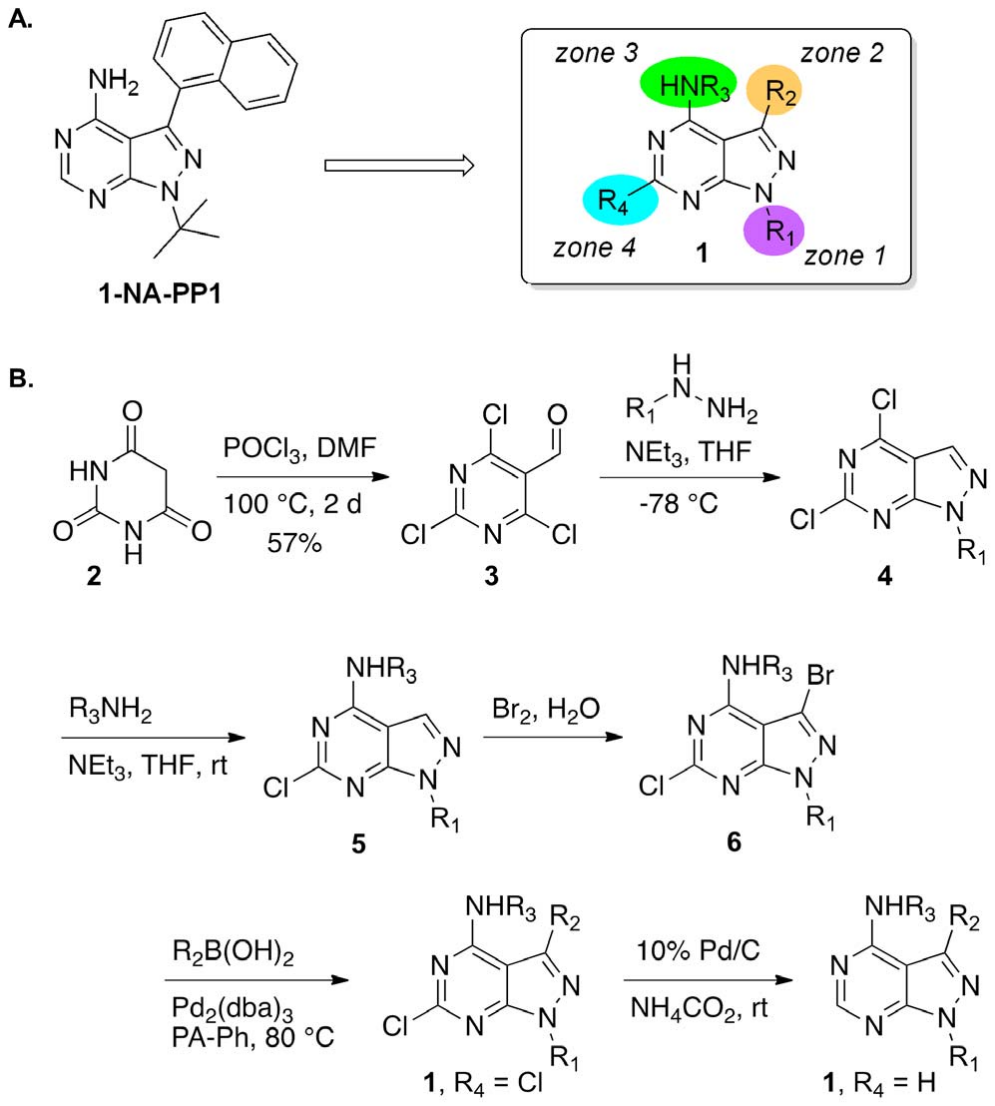
Synthesis and SAR analysis of 1-NA-PP1 analogs. **A**. 4-Zone model for 1-NA-PP1 analog synthesis. **B**. Synthesis of 1*H*-pyrazolo[3,4-*d*]pyrimidines **1**.

The goal of our synthetic studies was to modify the substituents on the 1-NA-PP1 pyrazolo[3,4-*d*]pyrimidine core structure and determine the biological response to these modifications, as well as to potentially identify more PKD subtype selective analogs. We considered 4 types of variations, as shown in **Fig. 4**, and prepared a total of 18 derivatives, including the resynthesized parent, 1-NA-PP1.

Only two variations were explored in zone 1, *t*-butyl and methyl (**Table 2**). At 1 µM concentration, 1-NA-PP1 inhibited PKD1 activity by 80%, whereas its R_1_ =  methyl analog **1f** only led to a 32% reduction of activity. All other analogs with a methyl substituent in zone 1 and various substitutions in zones 2–4 fared even worse and demonstrated <10% inhibitory activities at 1 µM concentrations. The requirement for bulky groups at R_1_ of the pyrazolopyrimidine has been previously recognized and appears to be a general feature for kinase inhibition with this scaffold [11]. However, even when maintaining the *t*-butyl group in zone 1, variations such as the introduction of a methyl group in zone 3 (**1a**), and replacements of the naphthyl substituent with other aryl rings (**1b–e**) ablated the PKD1 activity. Accordingly, our limited SAR highlighted 1-NA-PP1 as the most potent congener with very limited tolerance for structural modifications of substituents at the pyrazolopyrimidine core. Even electrophilic derivatives with a chlorine group in zone 4 and the potential for irreversible enzyme alkylation (**1j**, **1k**, **1m**, **1p**, **1r**) did not show an increase in potency. A similarly steep decrease in v-Src tyrosine kinase inhibitory activity of pyrazolopyrimidine derivatives has been noted previously and is likely related to a specific hydrogen bonding pattern in zones 2 and 3 in the ATP binding pocket that should not be perturbed [25]. Accordingly, we continued our investigation of the inhibitor profile of pyrazolopyrimidines on PKD with the most potent agent, 1-NA-PP1.

**Table 2:** Structures of 1-NA-PP1 analogs.

Entry	Compound	R_1_	R_2_	R_3_	R_4_
1	**1-NA-PP1**	*t*-butyl	1-naphthyl	H	H
2	**1a**	*t*-butyl	1-naphthyl	methyl	H
3	**1b**	*t*-butyl	(*p*-MeO)phenyl	H	H
4	**1c**	*t*-butyl	(*p*-MeO)phenyl	methyl	H
5	**1d**	*t*-butyl	(*p*-F)phenyl	H	H
6	**1e**	*t*-butyl	(*p*-Ph)phenyl	H	H
7	**1f**	methyl	1-naphthyl	H	H
8	**1g**	methyl	1-naphthyl	methyl	H
9	**1h**	methyl	(*p*-MeO)phenyl	H	H
10	**1i**	methyl	(*p*-MeO)phenyl	methyl	H
11	**1j**	methyl	(*p*-MeO)phenyl	H	Cl
12	**1k**	methyl	(*p*-MeO)phenyl	methyl	Cl
13	**1l**	methyl	(*p*-CF_3_O)phenyl	methyl	H
14	**1m**	methyl	(*p*-CF_3_O)phenyl	methyl	Cl
15	**1n**	methyl	(*p*-F)phenyl	methyl	H
16	**1o**	methyl	(*p*-F)phenyl	methyl	Cl
17	**1p**	methyl	(*p*-Ph)phenyl	methyl	H
18	**1q**	methyl	(*p*-Ph)phenyl	methyl	Cl

### 1-NA-PP1 is cell-active and causes target inhibition in prostate cancer cells

**Figure 5:**
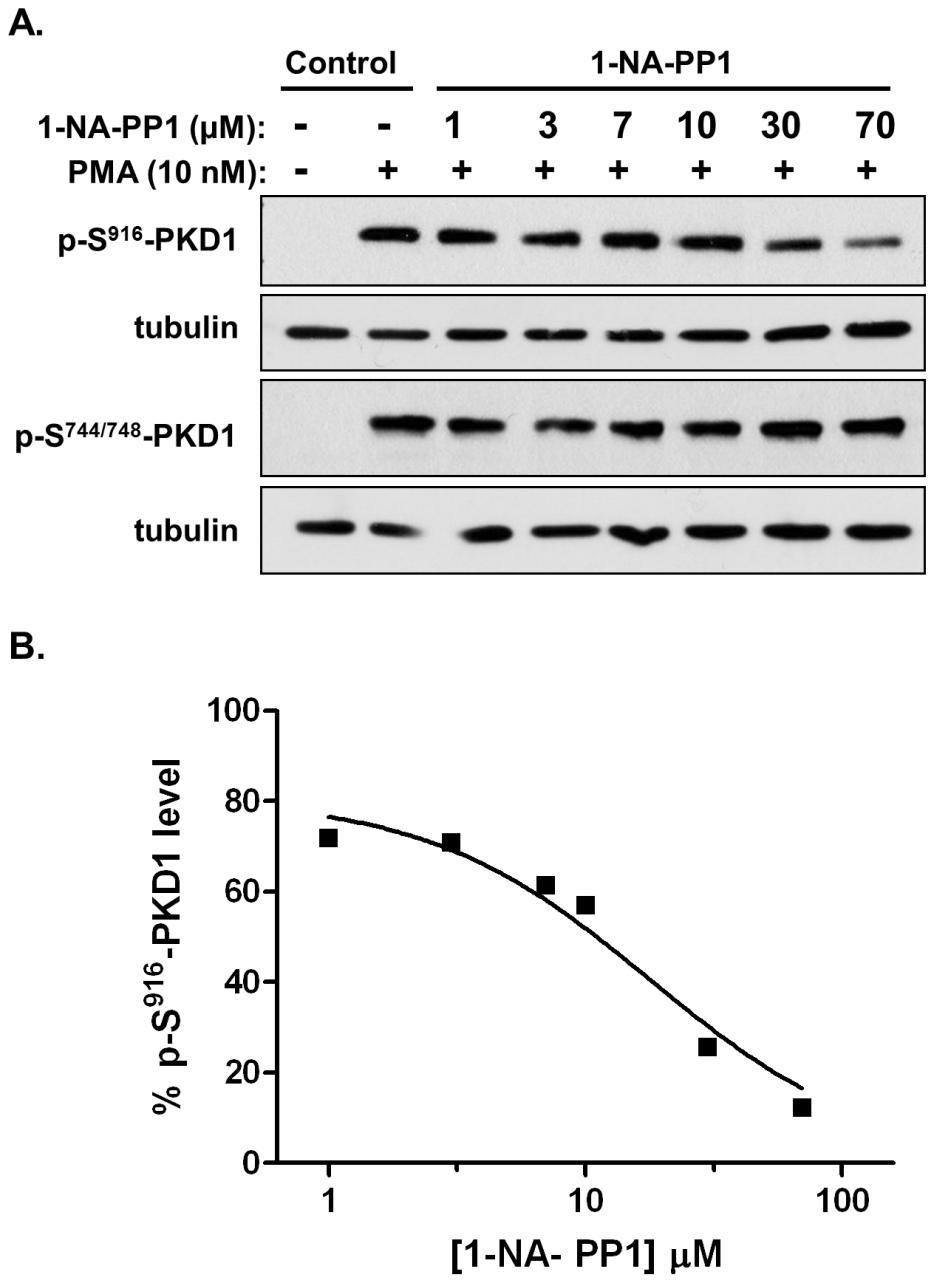
Inhibition of PMA-induced activation of endogenous PKD1 by 1-NA-PP1 in cells. **A**. LNCaP cells were pretreated with different doses of inhibitors for 45 min, followed by PMA stimulation at 10 nM for 20 min. Cell lysates were subjected to immunoblotting for p-S^916^-PKD1 and p-S^744/748^-PKD1. Tubulin was blotted as loading control. The experiment was repeated three times and the representative blots are shown. **B**. Determination of the IC_50_. Western blots were quantified using densitometry analysis. The data were plotted and IC_50_ values were derived the concentration-response curves using GraphPad. One of the three concentration-response curves was shown.

In this study, we examined whether 1-NA-PP1 was cell permeable and capable of target inhibition in intact cells. The effect of 1-NA-PP1 on 12-myristate 13-acetate (PMA)-induced endogenous PKD1 activation in LNCaP prostate cancer cells was examined as previously described [9], [23], [28]. PMA activates PKD through PKC-dependent trans-phosphorylation of Ser744/748 (S^744/748^) in the activation loop followed by autophosphorylation of PKD1 on Ser916 (S^916^) in the C-terminus [29], [30]. PKD1 activity correlates well with the level of phospho-S^916^ (p- S^916^) [30]. We therefore used p-S^916^ to monitor PKD activity and p-S^744/748^ of PKD1 to determine if the compound interfered with PKC-induced trans-phosphorylation by possibly inhibiting PKC. As shown in **Fig. 5**, treatment of LNCaP cells with 10 nM of PMA for 20 min induced both p-S^916^-PKD1 and p-S^744/748^-PKD1 signals. Pretreatment with increasing concentration of 1-NA-PP1 concentration-dependently inhibited autophosphorylation at p-Ser^916^-PKD1 with an IC_50_ of 22.5±1.5 µM (n = 2). In contrast, PKC-dependent trans-phosphorylation at Ser^744/748^ was unaffected by 1-NA-PP1. Thus, 1-NA-PP1 specifically abrogated PKD1 activity without blocking PKC-mediated trans-phosphorylation.

### 1-NA-PP1 potently blocks prostate cancer cell proliferation by inducing G2/M arrest

**Figure 6:**
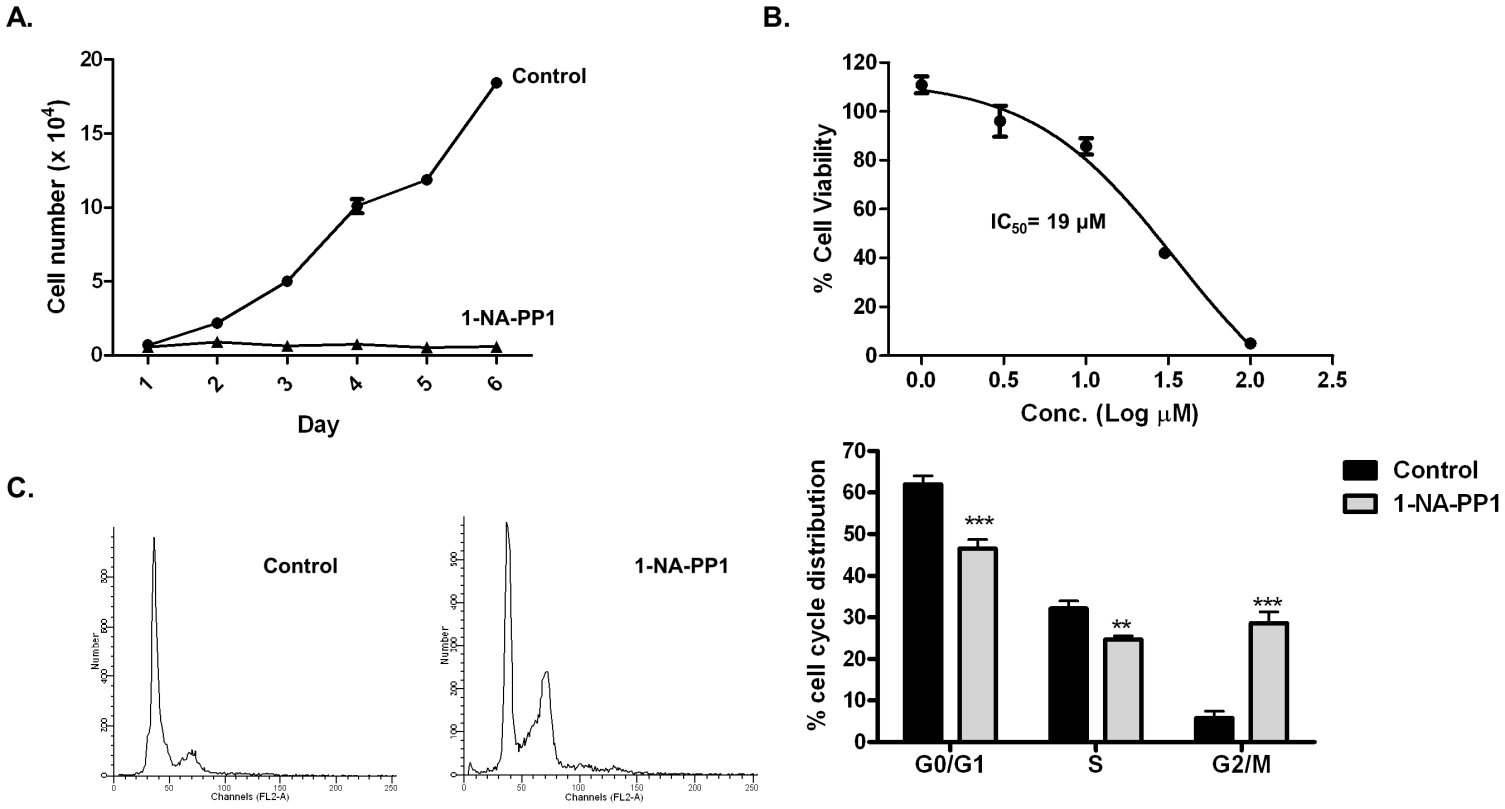
1-NA-PP1 inhibited PC3 cell proliferation, survival, and arrested cells in G2/M. **A.**1-NA-PP1 blocked PC3 cell proliferation. PC3 cells were plated in triplicates in 24-well plates. Cells were allowed to attach overnight. A cell count at day 1 was made, and then either a vehicle (DMSO) or 1-NA-PP1 at 10 µM was added. Cells were counted daily for a total of 5 days. Fresh media and inhibitor were added every 2 days. The means of triplicate determinations were plotted over time. The experiment was repeated twice and results from one representative experiment are shown. **B**. 1-NA-PP1 induced cell death in PC3 cells. PC3 cells were seeded into 96-well plates (3000 cells/well) and were then incubated in media containing 0.3–100 µM inhibitors for 72 h. MTT solution was added to each well and incubated for 4 h. Optical density was read at 570 nm to determine cell viability. The IC_50_ was determined as the mean of two independent experiments for each compound. **C.** 1-NA-PP1 caused G2/M phase cell cycle arrest. PC3 cells were treated with either vehicle (DMSO), or 10 µM 1-NA-PP1 for 48 h. Cell cycle distribution was determined by flow cytometry after propidium iodide labeling of fixed cells. Statistical significance was determined by unpaired t-test and is indicated. **, *p*<0.01; ***, *p*<0.001.

PKD has emerged as a promising therapeutic target for cancer. Here, the effects of targeted inhibition of PKD by 1-NA-PP1 on prostate cancer cell proliferation, survival, and cell cycle progression were examined. Cell proliferation was determined by treating the cells at 30 µM concentration of agent, and then cell number was counted for six consecutive days in the presence and absence of inhibitor. The growth and cytotoxic effects of 1-NA-PP1 were evaluated by cell number counts and MTT assay. As shown in **Fig. 6A**, 1-NA-PP1 at 30 µM caused drastic growth arrest of PC3 prostate cancer cells starting at day 2 and persisted to the end of the experiment (day 6). 1-NA-PP1 also concentration-dependently induced cell death with an IC_50_ of 23.3±5.7 µM (n = 3) (**Fig. 6B**). To provide insight in the effect of 1-NA-PP1 on cell proliferation, we examined the consequence of 1-NA-PP1 treatment on cell cycle distribution using flow cytometry. Cell cycle analysis was conducted after treating PC3 cells with 30 µM of 1-NA-PP1 for 72 h. As shown in **Fig. 6C**, 1-NA-PP1 significantly increased the proportion of cells in the G2/M phase of the cell cycle, corresponding to a shift from 5.8% cells in G2/M in the control to 28.6% in 1-NA-PP1-treated cells, and thus implying that the growth inhibition caused by 1-NA-PP1 is due to its ability to induce G2/M arrest.

### 1-NA-PP1-indued growth arrest is mediated through targeted inhibition of PKD

**Figure 7:**
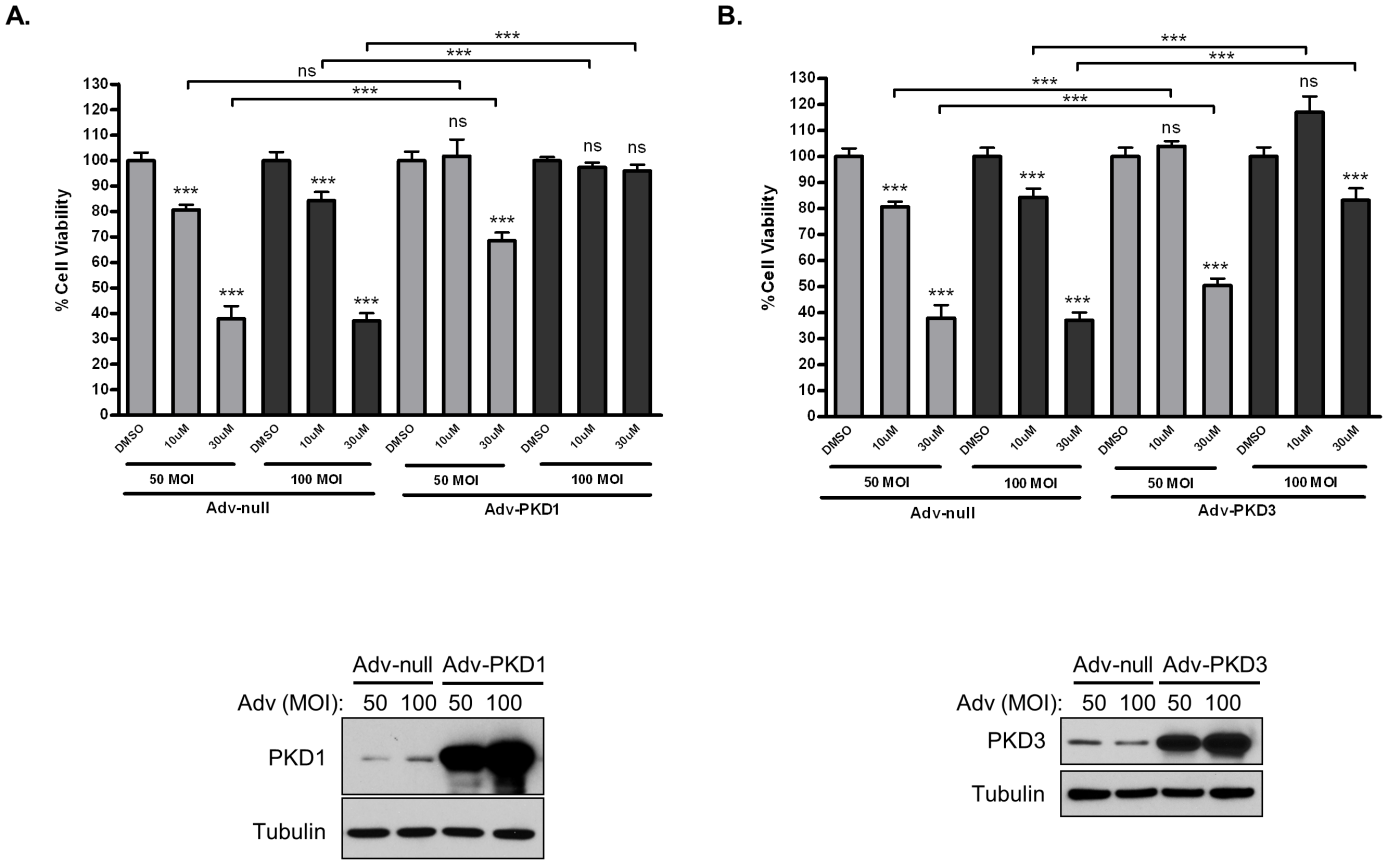
1-NA-PP1-induced growth arrest was mediated through targeted inhibition of PKD. Overexpression of PKD1 and PKD3 in prostate cancer cells rescued the anti-proliferative effects of 1-NA-PP1. PC3 (0.5 million) cells were seeded in a 60 mm dish and infected the next day with 50 and 100 MOI adenoviruses carrying PKD1 (Adv-PKD1) (**A**) and (Adv-PKD3) PKD3 (**B**). Empty adenovirus (Adv-null) was used as control. After 24 h, 3000 cells/well were plated in 96-well plates and treated with and without 10 and 30 µM 1-NA-PP1 for 72 h. MTT solution was added to each well and incubated for 4 h. Optical density was read at 570 nm to determine cell viability. The overexpression of PKD1 and PKD3 was confirmed by Western blotting analysis (images below the graphs). Statistical significance between DMSO and inhibitor treatment for each adenovirus as well as between control and PKD adenoviruses at each inhibitor concentration were determined by unpaired t-test in GraphPad Prism V. ns, not statistically significant; *, p<0.05; **, p<0.01; ***, p<0.001

To ensure the success of a targeted therapy, it is important to demonstrate target specificity at the biological level. Our previous data showed that the biological effects induced by PKD inhibitors phenocopied those caused by knockdown PKD isoforms, indicating that the effects of the inhibitors was mediated through targeted inhibition of PKD [9], [23]. In this study, we took a more direct approach to determine if a targeted inhibition of PKD accounts for the biological actions of 1-NA-PP1. Specifically, focusing on the anti-proliferative effect of 1-NA-PP1, we sought to determine if overexpression of PKD1 and PKD3 using adenoviruses could rescue the anti-proliferative effects of 1-NA-PP1. As shown in **Fig. 7A–B**, PC3 cells were infected with null adenovirus (Adv-null) and adenovirus carrying PKD1 and PKD3 genes (Adv-PKD1 and Adv-PKD3) at 50 and 100 MOI. The infected cells were subjected to 1-NA-PP1 treatment at 10 and 30 µM. Infection with Adv-PKD1 and Adv-PKD3 reversed the anti-proliferative effects of 1-NA-PP1. The higher levels of expression of PKD1 or PKD3, the greater the rescue effects, and at 100 MOI a nearly complete reversal of 1-NA-PP1-induced inhibition of cell proliferation was observed for Adv-PKD1. These data indicate that the anti-proliferative effects of 1-NA-PP1 were mediated through the inhibition of PKD. It also suggests that the functions of PKD isoforms are likely redundant since both PKD1 and PKD3 can rescue the effects of 1-NA-PP1.

### 1-NA-PP1 potently inhibits prostate tumor cell migration and invasion

**Figure 8:**
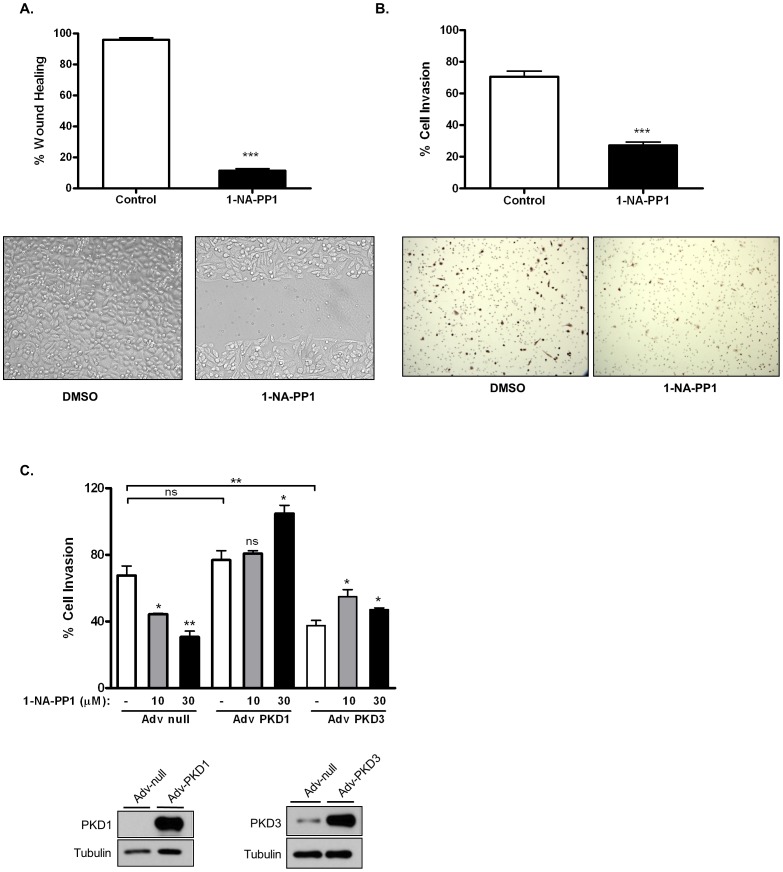
1-NA-PP1 blocked prostate cancer cell migration and invasion. **A.**1-NA- PP1 blocked prostate cancer cell migration. PC3 cells were grown to confluence in 6-well plates. Monolayer was wounded and imaged immediately (0 h). Cells were then treated in growth media containing a vehicle (DMSO) or 30 µM of 1-NA-PP1 for 22 h and wound closure was measured. Percentage wound healing was calculated as the percent of healed wound area as compared to the original wound. **B.** 1-NA-PP1 inhibited prostate cancer cell invasion. DU145 cells were incubated with 30 µM 1-NA-PP1 in Matrigel inserts. After 20 h, noninvasive cells were removed and invasive cells were fixed in 100% methanol, stained in 0.4% hematoxylin solution, and photographed. The number of cells that invaded the Matrigel matrix was determined by cell counts in 6 fields relative to the number of cells that migrated through the control insert. Percentage invasion was calculated as the percent of the cells invaded through Matrigel inserts vs. the total cells migrated through the control inserts. **C.** Overexpressed PKD1 and PKD3 reversed the inhibitory effects of 1-NA-PP1 on tumor cell invasion. DU145 cells were infected with null, PKD1, and PKD3 adenoviruses (Adv-null, Adv-PKD1, and Adv-PKD3) at 100 MOI. After 24 h, cells were replated in control and Matrigel inserts, and a Matrigel invasion assay was conducted as described above. The overexpression of PKD1 and PKD3 was confirmed by Western blotting analysis (images below the graphs). All the above experiments were repeated at least three times and data from a representative experiment are shown.

PKD has been shown to play an important role in the regulation of cell motility, adhesion and invasion [4]. In this study, the effects of 1-NA-PP1 on tumor cell migration and invasion were assessed by two independent assays, a wound healing assay (cell migration) and a Matrigel invasion assay (cell invasion). As illustrated in **Fig. 8A**, 1-NA-PP1 at 30 µM potently blocked PC3 cell migration. Twenty-two hours after wounding, 88.6% of the wounded area in the 1-NA-PP1-treated monolayer remained open while that of the control had completely closed. Similarly, 1-NA-PP1 also significantly blocked tumor cell invasion. Treatment of cells with 30 µM 1-NA-PP1 for 20 h resulted in >60% inhibition of cell invasion compared with control (**Fig. 8B**). Taken together, 1-NA-PP1 is a potent inhibitor of prostate cancer cell migration and invasion.

To determine if PKD mediates the effects of 1-NA-PP1 on migration and invasion, rescue experiments were conducted to examine if overexpressed PKD1 and PKD3 could dampen the inhibitory effects of 1-NA-PP1. PC3 cells were infected with null adenovirus (Adv-null) and adenovirus carrying PKD1 and PKD3 genes (Adv-PKD1 and Adv-PKD3). The invasive property of the infected cells was measured by Matrigel invasion assay. As shown in **Fig. 8C**, overexpression of PKD1 or PKD3 completely reversed the inhibition of 1-NA-PP1 on cell invasion, despite their lack of inhibitory effects on cells invasion when expressed alone. These data imply that targeted inhibition of PKD mediates the anti-invasive effects of 1-NA-PP1. In contrast, since overexpression of PKD1 and PKD3 blocked the migration of PC3 cells, we were not able to measure significant changes in cell migration in the presence or absence of 1-NA-PP1 using wound healing assay (data not shown). Alternatively, we examined the migration of DU145 cells using the migration chambers. Our data showed that overexpression of PKD1 or PKD3 inhibited cell migration and did not affect the inhibitory effect of 1-NA-PP1 on cell migration (**Fig. S1**).

### A gatekeeper mutant of PKD1 is 12-fold more sensitive to the inhibition of 1-NA-PP1 in intact cells

**Figure 9:**
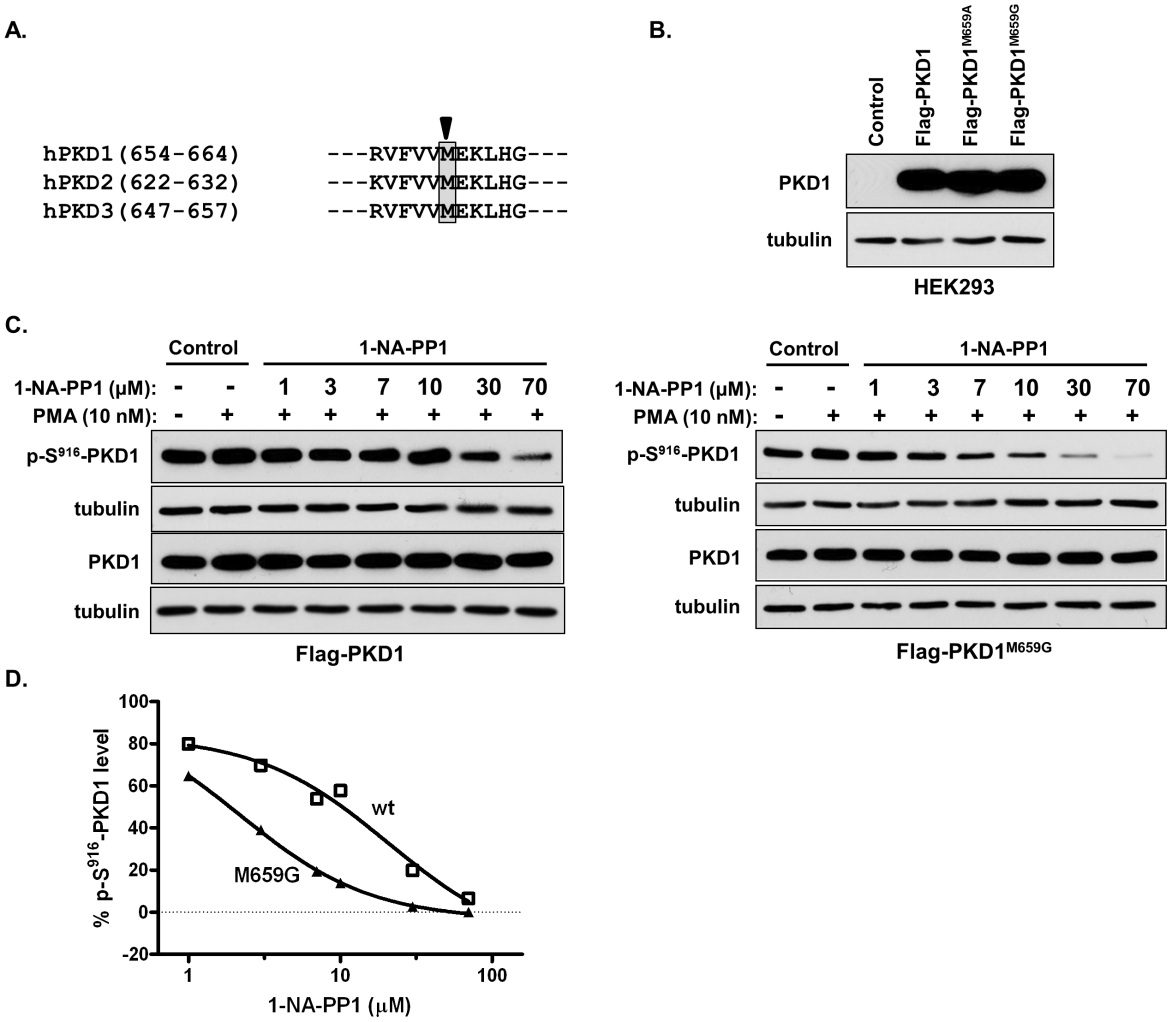
Mutating the gatekeeper amino acid sensitized PKD1 to the inhibition of 1-NA-PP1. **A.**Alignment of the primary sequences containing the gatekeeper amino acid in PKD. Arrow indicates the consensus gatekeeper amino acid “Methionine” (M) in a shaded rectangle. **B.** Expression of wild-type and mutant PKDs. HEK293 cells were transfected with wild-type and two gatekeeper mutants of Flag-PKD1 (Flag-PKD1^M659G^ and Flag-PKD1^M659A^). Two days after transfection, cells were lysed and subjected to Western blotting for PKD1 and tubulin (loading control). **C.** 1-NA-PP1 concentration-dependently inhibited PMA-induced activation of Flag-PKD1 and Flag-PKD1^M659G^. HEK293 cells transfected with Flag-PKD1 and Flag-PKD1^M659G^ were serum-starved for 24 h and pre-treated with 1-NA-PP1 at increasing concentrations in serum-free medium for 45 min, followed by stimulation with PMA at 10 nM for 20 min. The cells were harvested and subjected to immunoblotting for p-S^916^-PKD1, PKD1, and tubulin. The experiment was repeated three times and representative images from one experiment are shown.

1-NA-PP1 is one of the C3-modified analogs of the Src-family kinase inhibitor PP1. It was developed and utilized as a highly selective kinase inhibitor with single digit nanomolar IC_50_s for analog-sensitive (as) kinases that are engineered to carry a single amino acid substitution at the gatekeeper residue in the kinase active site [25], [27]. However, micromolar 1-NA-PP1 does not inhibit or only weakly blocks wild-type kinases, which allows the inhibitor/as-kinase pair to be used in dissecting the specific functions and signaling mechanisms of kinases. This chemical genetic approach has been successfully applied to a variety of protein kinases [27], [31]–[36]. Thus, we speculated that the potency and selectivity of 1-NA-PP1 for PKD could be further enhanced by introducing gatekeeper amino acid mutations. Alignment of the active site sequence of PKD isoforms led to the identification of the gatekeeper amino acid, methionine 659 (M659), in PKD1 (**Fig. 9A**). We subsequently generated two space-creating analog-sensitive PKD1 mutants of a Flag-tagged PKD1 (Flag-PKD1), namely Flag-PKD1^M659G^ and Flag-PKD1^M659A^. After overexpression in intact cells (**Fig. 9B**), the inhibitory activity of 1-NA-PP1 was assessed by pre-treatment with the inhibitor followed by PMA stimulation, and subsequent immunoblotting for p-S^916^-PKD1 as before (Fig. 5). PKD1 and tubulin were blotted as controls. As shown in **Fig. 9C**, Flag-PKD1^M659G^ demonstrated significantly increased sensitivity to 1-NA-PP1 as compared to the wild-type control. Based on the densitometry analysis, the IC_50_ for wild-type Flag-PKD1 was 26.50±2.23 µM, while the IC_50_ for the analog-sensitive Flag-PKD1^M659G^ was 2.13±0.61 µM, reflecting a 12-fold increase in sensitivity to 1-NA-PP1. In contrast, there was no significant difference in the inhibition of Flag-PKD1^M659A^ by 1-NA-PP1 as compared to the wild-type Flag-PKD1 (data not shown), indicating that the M659A mutation was unable to sensitize PKD1 to the inhibition of 1-NA-PP1. Taken together, our data indicate that the inhibitory activity of 1-NA-PP1 for PKD1 could be further enhanced by introducing the gatekeeper mutation, implying that 1-NA-PP1 could be paired with the analog-sensitive PKD1^M659G^ to determine PKD-specific functions and signaling pathways in intact cells.

## Discussion

PKDs play important roles in many fundamental biological processes and represent an emerging therapeutic target for many pathological conditions and diseases. However, the exact biological function of PKD has not been well defined. Highly selective and cell-permeable PKD small molecule inhibitors are not only potential drug candidates but also powerful tools for a systemic evaluation of PKD-specific functions and signaling pathways in complex biological systems. In this study, we report the identification and evaluation of a new cell-permeable PKD inhibitor, 1-NA-PP1. This pyrazolopyrimidine is an ATP-competitive pan-PKD inhibitor that blocked 50% of PKD activity at about 100 nM. At the cellular level, it inhibited PMA-induced endogenous PKD activation at about 20 µM without interfering with PKC-mediated trans-phosphorylation. Biologically, 1-NA-PP1 potently inhibited prostate cancer cell proliferation, migration and invasion, suggesting it is a promising antitumor agent for drug development.

The selectivity of 1-NA-PP1 was examined in a few closely related kinases including PKCα, PKCδ and CAMKIIα. 1-NA-PP1 did not significantly inhibit these kinases at concentrations up to 10 µM, which was in direct contrast to the promiscuous IKK-16 that was also identified in the targeted library screen. The exquisite selectivity of 1-NA-PP1 was further confirmed by its lack of inhibitory activity for PKC-dependent trans-phosphorylation at S^744/748^-PKD1, which excluded any possible interference with the upstream PKC activity in cells. 1-NA-PP1 was originally developed as a highly potent analog-sensitive (as) kinase inhibitor for Src and other kinases [25], [27]. A unique feature of this class of kinase inhibitors is that they are engineered to be poor inhibitors of the native wild-type kinases but potent and selective against the mutated analog-sensitive kinases, therefore ensuring high target-specificity of the compound on mutated kinases [25], [27]. This is largely due to the presence of the bulky naphthyl substituent which leads to a steric clash between this group and the side-chain of the gatekeeper amino acid in the active site. Mutation of the gatekeeper amino acid to space-creating small amino acids, such as glycine or alanine, results in a unique binding pocket that is thought to be sufficiently large to accommodate the naphthyl side-chain. Accordingly, 1-NA-PP1 has been shown to be a weak inhibitor of most native kinases, including the Src, Abl, CAMK, and CDK family of kinases [27], ERK1/2 [37], and PKA [32] (IC_50_>1 µM), providing further support for a high specificity for mutated kinases. The kinome scan data of 1-NM-PP1 (a close analog of 1-NA-PP1) against 300 protein kinases further support a highly selective profile of this inhibitor, with 22 out of 300 kinases was inhibited >50% and only three kinases (Ack1, CK1ε, EphA6) was inhibited >90% (note that these three kinases were among the most inhibited or promiscuous kinases in the 300 kinase panel) [26]. The kinome scan data also confirmed the inhibition of PKD isoforms and exclusive selectivity against PKC and CAMK family members. Additionally, we were able to demonstrate a 12-fold increase in sensitivity to 1-NA-PP1 for analog-sensitive (as) PKD1 mutant in intact cells as compared to wild-type PKD1. This finding provides the foundation for the use of a chemical genetic approach, e.g. by pairing of 1-NA-PP1 and PKD1^M659G^, to decipher PKD-specific functions and signaling pathways in different biological systems.

Among the 17 synthetic analogs of 1-NA-PP1 prepared in this work, only the R_1_ =  methyl analog **1f** led to a modest 32% reduction of PKD1 activity. Even conservative substitutions in zones 1–4 ablated the high level of activity observed for the parent compound. Electrophilic analogs for potential covalent attachment were also briefly investigated, but did not convey inhibitory effects. According, the SAR of 1-NA-PP1 is very narrow, and high activity in the PKD pocket requires a precise alignment of the optimal substituents on the pyrazolopyrimidine scaffold. This observation bodes well for the future use of 1-NA-PP1 in the development of PKD subtype-specific inhibitors.

In the past, anticancer activities have been demonstrated for several classes of PKD small molecule inhibitors. We have shown that CID755673 and its derivatives potently block prostate cancer cell proliferation, migration and invasion [23]. CRT0066101 has been demonstrated to inhibit pancreatic tumor growth and pancreatic tumor cell-induced angiogenesis *in vitro* and *in vivo* [5], [19]. In accordance with these findings, our data indicate that the new PKD inhibitor 1-NA-PP1 is also a potent anticancer agent in prostate cancer cells. It arrested prostate cancer cell proliferation in the G2/M phase of cell cycle, induced cell death, and blocked tumor cell migration and invasion. Its cytotoxic/growth inhibitory activity corresponded well with its inhibition of PKD activation in prostate cancer cells (∼20 µM). Meanwhile, this growth inhibitory effect could be reversed by overexpression of PKD1 or PKD3. These results strongly argue for the target specificity of 1-NA-PP1, indicating that the anti-proliferative effect of this compound was mediated through the inhibition of PKD. Aside from the efforts of using PKD siRNAs to phenocopy the biological effects induced by PKD inhibitors, this is the first direct evidence demonstrating the target specificity of a PKD inhibitor in intact cells. A similar approach was used to dampen or reverse the inhibitory effects of 1-NA-PP1 on cell migration and invasion. Although overexpression of PKD1 or PKD3 did not affect the inhibition of 1-NA-PP1 on cell migration, it completely reversed the anti-invasive activity of 1-NA-PP1, indicating that the anti-invasive effect was mediated through the inhibition of PKD. Interestingly, overexpression of PKD1 or PKD3 alone inhibited cell migration in our study, which is consistent with other reports demonstrating PKD as a negative regulator of directional cell migration through phosphorylation of the cofilin phosphates slingshot 1 like (SSH1L) [38], [39]. Clearly, the anti-migratory effect of 1-NA-PP1 is not mediated through the inhibition of PKD. Other likely targets of 1-NA-PP1 have been demonstrated (Table S1) and may account for this effect.

In summary, we have identified a new, highly selective, and cell-permeable PKD small molecule inhibitor, 1-NA-PP1. This compact pyrazolopyrimidine possesses potent antitumor activities in prostate cancer cells, thus suggesting its further development as a potential drug candidate. Additionally, this compound may be valuable for use in a chemical genetic approach with the analog-sensitive PKD to investigate PKD-specific functions and signaling mechanisms in diverse biological systems.

## Materials and Methods

### Chemicals and Reagents

Kinase active recombinant GST-tagged human protein kinase D1 (PKD1) was obtained from Enzo Life sciences (Farmingdale, NY). DMSO was purchased from Sigma. Recombinant PKCα, PKCδ and CAMKIIα were obtained from SignalChem (Richmond, BC, Canada). ATP was purchased from Fisher Scientific. HDAC5 substrate peptide was synthesized by Biobasic Canada Inc. (Markham, ON). Myelin basic protein 4–14 was purchased from AnaSpec Inc. (Fremont, CA). A pharmacologically active kinase inhibitor library was purchased from Tocris Bioscience (Minneapolis, MN).

### Synthesis of 1-NA-PP1 Analogs

Vilsmeier–Haack reaction of barbituric acid **2** provided the trichloropyrimidine **3** in 57% yield [40]. The pyrazole ring was closed by treatment with *t*-butyl or methyl hydrazine to give the pyrazolo[3,4-*d*]pyrimidine **4** [41]. Aminolysis with ammonia and methyl amine at room temperature provided the nucleophilic aromatic substitution product **5** [41] which was brominated to give the pyrazolopyrimidine **6** [40]. Attempts at forming the 3-iodo derivative with iodine [41], *N*-iodosuccinimide [42], or Barluenga's reagent [43] were not successful. A selective Suzuki coupling at the 3-position with aromatic boronic acids R_2_B(OH)_2_ led to the chlorinated derivative, and the chlorine group was reduced by a catalytic hydrogen transfer process to give product **1** with R_4_ = H (**Fig. 4B**). The experimental details and spectroscopic data on “The Synthesis of New Pyrazolopyrimidine Inhibitors of Protein Kinase D” were described in **File S1**.

### In Vitro Radiometric PKD1 Screening Assay

An *in vitro* radiometric kinase assay was used to screen an 80 compound library for PKD1 inhibitory activity at 1 µM concentration. 1.2 µM of a HDAC5 peptide [44] was used as substrate in the reaction. Phosphorylation of HDAC5 was detected in a kinase reaction having 1 µCi [γ-^32^P] ATP (Perkin Elmer Life Sciences), 25 µM ATP, 50 ng purified recombinant PKD1 in 50 µL kinase buffer containing 50 mM Tris-HCl, pH 7.5, 4 mM MgCl_2_ and 10 mM β-mercaptoethanol. The reaction was incubated at 30°C for 10 minutes and 25 µL of the reaction was spotted on Whatman P81 filter paper. The filter paper was washed 3 times in 0.5% phosphoric acid, air dried and counted using Beckman LS6500 multipurpose scintillation counter. Percent PKD1 inhibition was graphed using GraphPad Prism software 5.0.

### In Vitro Radiometric PKC and CAMKIIα Kinase Assay

The PKC kinase assay was carried out by co-incubating 1 µCi [γ-^32^P]ATP, 20 µM ATP, 50 ng of purified PKCα or PKCδ and 5 µg of myelin basic protein 4–14, 0.25 mg/mL bovine serum albumin, 0.1 mg/mL phosphatidylcholine/phosphatidylserine (80/20%) (1 µM), 1 µM phorbol dibutyrate in 50 µL of kinase buffer containing 50 mM Tris-HCl, pH 7.5, 4 mM MgCl_2_ and 10 mM β-mercaptoethanol. For the CAMK assay, 50 ng of CAMKIIα and 2 µg syntide-2 substrate in 50 µL kinase buffer were incubated with 0.1 mM MgCl_2_, 1 µCi of [γ-^32^P] ATP, 70 µM ATP. 0.5 mM CaCl_2_ and 30 ng/µL calmodulin were preincubated for 15 min on ice and then added in the kinase reaction. The reactions were incubated at 30°C for 10 min and 25 µL of the reaction was spotted on Whatman P81 filter paper. The filter paper was washed 3 times in 0.5% phosphoric acid, air dried and counted using Beckman LS6500 multipurpose scintillation counter.

### Cell Lines and Western Blot Analysis

The prostate cancer cell lines (LNCaP, DU145, and PC3) were obtained from the American Type Culture Collection (ATCC). LNCaP and DU145 prostate cancer cells were maintained in RPMI 1640, while PC3 cells were maintained in Ham's F-12 medium, supplemented with 10% fetal bovine serum (FBS) and 1000 units/L penicillin, and 1 mg/mL streptomycin in 5% CO_2_ at 37°C. Western blot analysis was carried out as previously reported [9]. Briefly, cells were lysed in buffer containing 200 mM Tris-HCl, pH 7.4, 100 µM 4-(2-aminoethyl) benzenesulfonyl fluoride, 1 mM EGTA, and 1% Triton X-100. Protein concentration was determined using the BCA Protein Assay kit (Pierce) and then equal amounts of protein were subjected to SDS-PAGE followed by electrotransfer to nitrocellulose membranes. Membranes were blocked with 5% nonfat milk in Tris-buffered saline and then probed with primary antibodies for either p-S916-PKD1 (Millipore), p-S744/748-PKD1, PKD (Cell Signaling Technology), and tubulin (Sigma), followed by anti-mouse or anti-rabbit secondary antibodies conjugated to horseradish peroxidase (Bio-Rad). The enhanced chemiluminescence (ECL) Western blotting detection system (Pierce) was used to facilitate detection of protein bands.

### MTT Assay

PC3 cells were seeded into 96-well plates (3000 cells/well) and allowed to attach overnight. Cells were then incubated in media containing 0.7–100 µM inhibitors for 72 h. 3-(4,5-Dimethylthiazol-2-yl)-2,5-diphenyltetrazolium bromide methyl thiazolyl tetrazolium (MTT) solution was prepared at 2 mg/mL concentration in PBS, sterilized by filtering through a 0.2 µm filter, and wrapped in foil to protect from light. 50 µL MTT solution was added to each well and incubated for 4 h at 37°C. Then, media was removed and 200 µL DMSO was added to each well. The plate was mix by shaking for 5 min and the optical density was determined at 570 nm.

### Cell Proliferation Assay and Cell Cycle Analysis

Proliferation of PC3 cells was measured by counting the number of viable cells upon trypan blue staining as previously described [9]. Cell cycle analysis was performed as described [23]. Briefly, PC3 cells were treated with indicated compounds at 30 µM for 72 h, and then fixed in 70% ice-cold ethanol overnight, followed by labeling with propidium iodide. The labeled cells were analyzed using a FACSCalibur flow cytometer (BD Biosciences).

### Wound Healing Assay

PC3 or DU145 cells were grown to confluence in 6-well plates. Migration was initiated by scraping the monolayer with a pipette tip, creating a “wound.” The indicated concentration of compound was added to the media, and the wound was imaged immediately under an inverted phase-contrast microscope with 10× objective. After 24 h, a final image was taken. The wound gap was measured, and % wound healing was calculated. The average % wound healing was determined based on at least 6 measurements of the wound gap.

### Matrigel Invasion Assay

DU145 cells (4.0×10^4^ cells/ml) in RPMI containing 0.1% fetal bovine serum (FBS) were seeded into the top chamber of BioCoat control inserts (pore size 8 µm) or BioCoat Matrigel invasion inserts with Matrigel-coated filters (BD Pharmingen). To stimulate invasion, media in the lower chamber of the insert contained 20% FBS. Inhibitors were added at 30 µM concentration to both the upper and lower chambers, and cells were incubated for 22 h. After incubation, noninvasive cells were removed using a cotton swab, and invasive cells were fixed in 100% methanol and stained with 0.4% hematoxylin. After staining, cells were counted under a microscope (200× magnification). The percentage invasion was determined by cell counts in 5 fields of the number of cells that invaded the Matrigel matrix relative to the number of cells that migrated through the control insert.

## Supporting Information

Figure S1 Overexpressed PKD1 and PKD3 inhibited tumor cell migration. DU145 cells were infected with null, PKD1, and PKD3 adenoviruses (Adv-null, Adv-PKD1, and Adv-PKD3) at 100 MOI. After 24 h, cells were replated in migration chambers and incubated with vehicle or 30 µM 1-NA-PP1 for 20 h. The number of cells that migrated was determined by cell counts in 6 fields. Percentage migration was calculated as the percent of the Adv-null treated with vehicle DMSO (set to 100%). The experiment was repeated three times and data from a representative experiment are shown.
(TIF)

File S1 The Synthesis of New Pyrazolopyrimidine Inhibitors of Protein Kinase D. Experimental details and spectroscopic data for 1-NA-PP1 analogs.
(DOCX)

Table S1 The specificity of 1-NM-PP1 in a kinome scan. The activity of 1-NM-PP1, along with 178 known kinase inhibitors, was profiled against a panel of 300 recombinant human protein kinases at a concentration of 0.5 µM in the presence of 10 µM ATP. Data were extracted at a cut-off of <50% residual kinase activity from the Kinase Inhibitor Resource (KIR) online tool (http://kir.fccc.edu/). The kinase activity was determined using a radiometric HotSpot assay which directly measures kinase catalytic activity toward a specific substrate.
(DOCX)
