# Supplementary material for: New Pyrazolopyrimidine Inhibitors of Protein Kinase D as Potent Anticancer Agents for Prostate Cancer Cells
Source: PLoS One. 2013 Sep 23;8(9):e75601. doi: 10.1371/journal.pone.0075601 (PMC3781056; doi:10.1371/journal.pone.0075601)
Supplement: File S1 — The Synthesis of New Pyrazolopyrimidine Inhibitors of Protein Kinase D. Experimental details and spectroscopic data for 1-NA-PP1 analogs. (DOCX) [file pone.0075601.s002.docx]

**Supporting Information**

**The Synthesis of New Pyrazolopyrimidine Inhibitors of Protein Kinase D**

Manuj Tandon, James Johnson, Zhihong Li, Shuping Xu, Peter Wipf, Q. Jane Wang

**Table of Contents**

General Experimental S2

1-(*t*-Butyl)-3-(naphthalen-1-yl)-1*H*-pyrazolo[3,4-*d*]pyrimidin-4-amine (**1-NA-PP1**) S3

1-(*t*-Butyl)-*N*-methyl-3-(naphthalen-1-yl)-1*H*-pyrazolo[3,4-*d*]pyrimidin-4-amine (**1a**) S4

1-(*t*-Butyl)-3-(4-methoxyphenyl)-1*H*-pyrazolo[3,4-*d*]pyrimidin-4-amine (**1b**) S5

1-(*t*-Butyl)-3-(4-methoxyphenyl)-*N*-methyl-1*H*-pyrazolo[3,4-*d*]pyrimidin-4-amine (**1c**) S6

1-(*t*-Butyl)-3-(4-fluorophenyl)-1*H*-pyrazolo[3,4-*d*]pyrimidin-4-amine (**1d**) S7

3-([1,1'-Biphenyl]-4-yl)-1-(*t*-butyl)-1*H*-pyrazolo[3,4-*d*]pyrimidin-4-amine (**1e**) S8

1-Methyl-3-(naphthalen-1-yl)-1*H*-pyrazolo[3,4-*d*]pyrimidin-4-amine (**1f**) S9

*N*,1-Dimethyl-3-(naphthalen-1-yl)-1*H*-pyrazolo[3,4-*d*]pyrimidin-4-amine (**1g**) S10

3-(4-Methoxyphenyl)-1-methyl-1*H*-pyrazolo[3,4-*d*]pyrimidin-4-amine (**1h**) S11

3-(4-Methoxyphenyl)-*N*,1-dimethyl-1*H*-pyrazolo[3,4-*d*]pyrimidin-4-amine (**1i**) S11

6-Chloro-3-(4-methoxyphenyl)-1-methyl-1*H*-pyrazolo[3,4-*d*]pyrimidin-4-amine (**1j**) S12

6-Chloro-3-(4-methoxyphenyl)-*N*,1-dimethyl-1*H*-pyrazolo[3,4-*d*]pyrimidin-4-amine (**1k**)

S13

*N*,1-Dimethyl-3-(4-(trifluoromethoxy)phenyl)-1*H*-pyrazolo[3,4-*d*]pyrimidin-4-amine (**1l**)

S13

6-Chloro-*N*,1-dimethyl-3-(4-(trifluoromethoxy)phenyl)-1*H*-pyrazolo[3,4-*d*]pyrimidin-4-amine (**1m**) S14

3-(4-Fluorophenyl)-*N*,1-dimethyl-1H-pyrazolo[3,4-d]pyrimidin-4-amine (**1n**) S15

6-Chloro-3-(4-fluorophenyl)-*N*,1-dimethyl-1*H*-pyrazolo[3,4-*d*]pyrimidin-4-amine (**1o**) S15

3-([1,1'-Biphenyl]-4-yl)-*N*,1-dimethyl-1*H*-pyrazolo[3,4-*d*]pyrimidin-4-amine (**1p**) S16

3-([1,1'-Biphenyl]-4-yl)-6-chloro-*N*,1-dimethyl-1*H*-pyrazolo[3,4-*d*]pyrimidin-4-amine (**1q**)

S17

2,4,6-Trichloropyrimidine-5-carbaldehyde (**3**) S17

4,6-Dichloro-1-methyl-1*H*-pyrazolo[3,4-*d*]pyrimidine (**4a**) S18

1-(*t*-Butyl)-4,6-dichloro-1*H*-pyrazolo[3,4-*d*]pyrimidine (**4b**) S18

6-Chloro-*N*,1-dimethyl-1*H*-pyrazolo[3,4-*d*]pyrimidin-4-amine (**5a**) S19

6-Chloro-1-methyl-1*H*-pyrazolo[3,4-*d*]pyrimidin-4-amine (**5b**) S19

1-(*t*-Butyl)-6-chloro-1*H*-pyrazolo[3,4-*d*]pyrimidin-4-amine (**5c**) S20

1-(*t*-Butyl)-6-chloro-*N*-methyl-1*H*-pyrazolo[3,4-*d*]pyrimidin-4-amine (**5d**) S20

3-Bromo-6-chloro-*N*,1-dimethyl-1*H*-pyrazolo[3,4-*d*]pyrimidin-4-amine (**6a**) S21

3-Bromo-6-chloro-1-methyl-1*H*-pyrazolo[3,4-*d*]pyrimidin-4-amine (**6b**) S21

3-Bromo-1-(*t*-butyl)-6-chloro-1*H*-pyrazolo[3,4-*d*]pyrimidin-4-amine (**6c**) S22

3-Bromo-1-(*t*-butyl)-6-chloro-*N*-methyl-1*H*-pyrazolo[3,4-*d*]pyrimidin-4-amine (**6d**) S22

References S23

**General Experimental**. Moisture and air-sensitive reactions were performed under N_2_ or Ar atmosphere and glassware used for these reactions was flamed dried and cooled under N_2_ or Ar prior to use. THF and Et_2_O were distilled from sodium/benzophenone ketyl. DMF and CHCl_3_ were distilled from CaH_2_. 1,4-Dioxane was purchased from Acros (Sure/Seal bottle) and used as received. Triethylamine was distilled from CaH_2_ and stored over KOH. Toluene was purified by passage through an activated alumina filtration system. Methylamine hydrochloride was recrystallized using absolute ethanol. 1,3,5,7-Tetramethyl-6-phenyl-2,4,8-trioxa-6-phosphaadamantane (PA-Ph) was obtained from Sigma Aldrich and used as received. All other materials were obtained from commercial sources and used as received. Melting points were determined using Mel-Temp II. Infrared spectra were determined using a Smiths Detection IdentifyIR FT-IR spectrometer. High-resolution mass spectra were obtained on a Micromass UK Limited, Q-TOF Ultima API, or Thermo Scientific Exactive Orbitrap LC-MS. Automated column chromatography was done using Isco Combiflesh Rf. ^1^H and ^13^C NMR spectra were obtained on Bruker Avance 300 MHz, 400 MHz, 500 MHz or 700 MHz instruments. Chemical shifts (δ) were reported in parts per million with the residual solvent peak used as an internal standard, δ ^1^H/^13^C (Solvent): 7.26/77.00 (CDCl_3_); 2.04/29.80 (acetone-d6); 2.50/39.52 (DMSO-d6); and are tabulated as follows: chemical shift, multiplicity (s = singlet, bs = broad singlet, d = doublet, bd = broad doublet, t = triplet, app t = apparent triplet, q = quartet, m = multiplet), coupling constant(s), and number of protons. ^13^C NMR spectra were obtained at 75 MHz, 100 MHz, 125 MHz, or 175 MHz using a proton-decoupled pulse sequence and are tabulated by observed peak. CDCl_3_ was filtered through dried basic alumina prior to sample preparation. Thin-layer chromatography was performed using pre-coated silica gel 60 F_254_ plates (EMD, 250 μm thickness) and visualization was accomplished with a 254 nm UV light and by staining with a phosphomolybdic acid solution or Vaughn’s reagent. Chromatography on SiO_2_ (Silicycle, Silia-P Flash Silica Gel or SiliaFlash® P60, 40-63 μm) was used to purify crude reaction mixtures. Final products were >95% purity as analyzed by reverse-phase HPLC (Alltech Prevail C-18, 100 × 4.6 mm, 1 mL/min, CH_3_CN, H_2_O and 0.1% TFA) with UV (210, 220 and 254 nm), ELS (nebulizer 45 ^o^C, evaporator 45 ^o^C, N_2_ flow 1.25 SLM), and MS detection using a Thermo Finnigan Surveyor LC, LCQ Advantage MS system (ESI positive mode), and a Thermo Scientific Exactive Orbitrap LC-MS (ESI positive).

**1-(*tert*-Butyl)-3-(naphthalen-1-yl)-1*H*-pyrazolo[3,4-*d*]pyrimidin-4-amine (1-NA-PP1)**.^1,2,3^ To a solution of dichloro[1,1'-bis(diphenylphosphino)ferrocene]palladium(II)-dichloromethane adduct (18.2 mg, 0.0200 mmol) in freshly distilled and degassed 1,4-dioxane (2 mL) was added 3-bromo-1-(*t*-butyl)-6-chloro-1*H*-pyrazolo[3,4-*d*]pyrimidin-4-amine (**6c**, 75.0 mg, 0.250 mmol), Cs_2_CO_3_ (122 mg, 0.370 mmol) and 1-naphthylboronic acid (50.0 mg, 0.300 mmol). The flask was sealed and the reaction mixture was stirred at 100 ^o^C for 2 d, filtered through Celite and concentrated under reduced pressure. The residue was purified by column chromatography on SiO_2_ (hexanes:EtOAc, 6:1) to yield a mixture of 1-(*t*-butyl)-6-chloro-3-(naphthalen-1-yl)-1*H*-pyrazolo[3,4-*d*]pyrimidin-4-amine and starting material (3:1, 43.6 mg) as an off white solid.

A solution of this mixture (40.0 mg, 0.0825 mmol), 5% Pd/C (1.08 g, 0.460 mmol) and NH_4_HCO_2_ (43.0 mg, 0.680 mmol) in MeOH (8 mL) was stirred at rt for 10 min, purged with H_2_ for 30 min and stirred under H_2_ overnight. The reaction mixture was filtered through Celite, which was rinsed with CH_2_Cl_2_ (10 mL), and the combined filtrate was concentrated under reduced pressure. The residue was purified by chromatography on SiO_2_ (hexanes:EtOAc, 4:1) to yield **1-NA-PP1** (5.90 mg, 23%, 95% purity by LC-MS) as a colorless solid: ^1^H NMR (CDCl_3_, 400 MHz) δ 8.39 (bs, 1 H), 7.98 (d, *J* = 8.0 Hz, 1 H), 7.94 (t, *J* = 6.6 Hz, 1 H), 7.67 (d, *J* = 6.6 Hz, 1 H), 7.61 (d, *J* = 8.0 Hz, 1 H), 7.85-7.49 (m, 2 H), 5.00 (bs, 2 H), 1.89 (s, 9 H); ^13^C NMR (CDCl_3_, 100 MHz) δ 157.6, 154.7, 153.9, 134.0, 131.8, 130.6, 129.6, 128.4, 128.4, 127.1, 126.5, 125.6, 125.5, 60.6, 29.3; HRMS (ESI) *m/z* calcd for C_19_H_19_N_5_ (M+H)^+^ 318.1719, found 318.1698.

**1-(*tert*-Butyl)-*N*-methyl-3-(naphthalen-1-yl)-1*H*-pyrazolo[3,4-*d*]pyrimidin-4-amine (1a)**.^1,2^ To a solution of dichloro[1,1'-bis(diphenylphosphino)ferrocene]palladium (II)-dichloromethane adduct (17.4 mg, 0.0200 mmol) in freshly distilled and degassed 1,4-dioxane (2 mL) was added 3-bromo-1-(*t*-butyl)-6-chloro-*N*-methyl-1*H*-pyrazolo[3,4-*d*]pyrimidin-4-amine (**6d**, 75.0 mg, 0.240 mmol) and Cs_2_CO_3_ (116 mg, 0.350 mmol) and the resulting mixture was stirred for 10 min, and treated with 1-naphthylboronic acid (50.0 mg, 0.280 mmol). The flask was sealed, heated to 100 ^o^C overnight, and the reaction mixture was filtered through Celite. The filtrate was concentrated under reduced pressure. The residue was purified by automated chromatography on SiO_2_ (12 g column, stepwise gradient, hexanes to EtOAc over 15 min) to yield a mixture of 1-(*t*-butyl)-6-chloro-*N*-methyl-3-(naphthalen-1-yl)-1*H*-pyrazolo[3,4-*d*]pyrimidin-4-amine and starting material (3:1, 30.0 mg) as an off-white solid.

A solution of this mixture (31.8 mg, 0.0652 mmol), 10% Pd/C (15.4 mg, 0.130 mmol) and NH_4_HCO_2_ (32.8 mg, 0.521 mmol) in MeOH (5 mL) was stirred at rt for 30 min, purged with H_2_ for 15 min and stirred under H_2_ overnight. The reaction mixture was filtered through Celite and the filtrate was concentrated under reduced pressure. The residue was purified by preparative TLC on SiO_2_ (hexanes:EtOAc, 5:1) to yield **1a** (4.85 mg, 23%, 100% purity by LC-MS) as a clear oil which crystallized after ~1 h: Mp 152.4-156.3 °C; IR (neat) 3437, 3044, 2975, 2926, 1597, 1558, 1312, 1243, 1079, cm^-1^; ^1^H NMR (CDCl_3_, 400 MHz) δ 8.47 (s, 1 H), 7.97 (dd, *J* = 14.4, 8.0 Hz, 2 H), 7.90 (d, *J* = 8.0 Hz, 1 H), 7.65-7.50 (m, 4 H), 4.71 (bs, 1 H), 2.86 (d, *J* = 4.8 Hz, 3 H), 1.88 (s, 9 H); ^13^C NMR (CDCl_3_, 100 MHz) δ 157.6, 154.7, 153.3, 139.7, 134.1, 131.9, 129.5, 128.4, 128.3, 127.0, 126.5, 125.7, 125.5, 101.7, 60.4, 29.3, 27.6; HRMS (ESI) *m/z* calcd for C_20_H_21_N_5_ (M+H)^+^ 332.1875, found 332.1863.

**1-(*t*-Butyl)-3-(4-methoxyphenyl)-1*H*-pyrazolo[3,4-*d*]pyrimidin-4-amine (1b)**.^1,2,3^ To a solution of dichloro[1,1'-bis(diphenylphosphino)ferrocene]palladium (II)-dichloromethane adduct (18.0 mg, 0.0200 mmol) in freshly distilled and degassed 1,4-dioxane (2 mL) was added 3-bromo-1-(*t*-butyl)-6-chloro-1*H*-pyrazolo[3,4-*d*]pyrimidin-4-amine (**6c**, 75.0 mg, 0.250 mmol) and Cs_2_CO_3_ (122 mg, 0.370 mmol). The reaction mixture was stirred for 10 min and 4-methoxybenzeneboronic acid (45.0 mg, 0.300 mmol) was added. The flask was sealed and the solution was stirred at 100 ^o^C overnight, filtered through Celite and concentrated under reduced pressure. The residue was purified by automated chromatography on SiO_2_ (4 g column, stepwise gradient, hexanes to EtOAc over 15 min) to yield a mixture of 1-(*t*-butyl)-6-chloro-3-(4-methoxyphenyl)-1*H*-pyrazolo[3,4-*d*]pyrimidin-4-amine and starting material (20:1, 48.0 mg) as an off white solid.

A solution of this mixture (20.0 mg, 0.0571 mmol), 10% Pd/C (85.7 mg, 0.0700 mmol) and NH_4_HCO_2_ (22.8 mg, 0.360 mmol) in MeOH (2 mL) was stirred at rt for 30 min, purged with H_2_ for 15 min and stirred under H_2_ overnight. The reaction mixture was filtered through Celite and the filtrate was concentrated under reduced pressure. The residue was purified by chromatography on SiO_2_ (hexanes:EtOAc, 6:1) to yield **1b** (14.0 mg, 85%, 96% purity by LC-MS) as a colorless solid: ^1^H NMR (CDCl_3_, 400 MHz) δ 8.34 (s, 1 H), 7.61 (d, *J* = 8.6 Hz, 1 H), 7.04 (d, *J* = 8.6 Hz, 1 H), 5.51 (bs, 2 H), 3.87 (s, 3 H), 1.83 (s, 9 H); ^13^C NMR (CDCl_3_, 100 MHz) δ 160.1, 157.8, 154.4, 154.1, 141.9, 129.9, 126.1, 114.7, 99.7, 60.3, 55.4, 29.2; HRMS (ESI) *m/z* calcd for C_16_H_19_N_5_O (M+H)^+^ 298.1668, found 298.1649.

**1-(*t*-Butyl)-3-(4-methoxyphenyl)-*N*-methyl-1*H*-pyrazolo[3,4-*d*]pyrimidin-4-amine (1c)**.^1,2^ To a solution of dichloro[1,1'-bis(diphenylphosphino)ferrocene]palladium (II)-dichloromethane adduct (1.60 mg, 0.0157 mmol) in freshly distilled and degassed 1,4-dioxane (2 mL) was added 3-bromo-1-(*t*-butyl)-6-chloro-*N*-methyl-1*H*-pyrazolo[3,4-*d*]pyrimidin-4-amine (**6d**, 50.0 mg, 0.157 mmol), and Cs_2_CO_3_ (77.4 mg, 0.235 mmol). The resulting mixture was stirred for 10 min and 4-methoxybenzeneboronic acid (28.6 mg, 0.188 mmol) was added. The flask was sealed and heated at 80 ^o^C overnight and at 100 ^o^C for an additional 4 h. The reaction mixture was filtered through Celite and the filtrate was concentrated under reduced pressure. The residue was purified by automated chromatography on SiO_2_ (4 g column, stepwise gradient, hexanes to EtOAc over 15 min) to yield a mixture of 1-(*t*-butyl)-6-chloro-3-(4-methoxyphenyl)-*N*-methyl-1*H*-pyrazolo[3,4-*d*]pyrimidin-4-amine and starting material (20:1, 27.5 mg) as an off white solid.

A solution of this mixture (14.5 mg, 0.0381 mmol), 10% Pd/C (7.44 mg, 0.0629 mmol) and NH_4_HCO_2_ (15.9 mg, 0.250 mmol) in MeOH (5 mL) was stirred at rt for 30 min, purged with H_2_ for 15 min and stirred under H_2_ overnight. The reaction mixture was filtered through Celite, and the filtrate was concentrated under reduced pressure. The residue was purified by chromatography on SiO_2_ (hexanes:EtOAc, 4:1) to yield **1c** (7.60 mg, 61%, 100% purity by LC-MS) as a clear oil which crystallized after ~1 h: Mp 89.3-91.2 °C; IR (neat) 3439, 2977, 2930, 1596, 1560, 1517, 1314, 1245, 1033 cm^-1^; ^1^H NMR (CDCl_3_, 400 MHz) δ 8.43 (s, 1 H), 7.57 (d, *J* = 8.8 Hz, 2 H), 7.05 (d, *J* = 8.8 Hz, 2 H), 5.29 (bd, *J* = 4.6 Hz, 1 H), 3.88 (s, 3 H), 3.21 (d, *J* = 4.6 Hz, 3 H), 1.82 (s, 9 H); ^13^C NMR (CDCl_3_, 100 MHz) δ 160.0, 157.8, 154.5, 153.5, 141.3, 129.9, 126.4, 114.7, 100.0, 60.1, 55.4, 29.2, 27.7; HRMS (ESI) *m/z* calcd for C_20_H_21_N_5_ (M+H)^+^ 312.1824, found 312.1814.

**1-(*tert*-Butyl)-3-(4-fluorophenyl)-1*H*-pyrazolo[3,4-*d*]pyrimidin-4-amine (1d)**.^1,2,3^ To a solution of dichloro[1,1'-bis(diphenylphosphino)ferrocene]palladium (II)-dichloromethane adduct (18.2 mg, 0.02 mmol) in freshly distilled and degassed 1,4-dioxane (2 mL) was added 3-bromo-1-(*t*-butyl)-6-chloro-1*H*-pyrazolo[3,4-*d*]pyrimidin-4-amine (**6c**, 75.0 mg, 0.250 mmol) and Cs_2_CO_3_ (122 mg, 0.370 mmol). The resulting mixture was stirred for 10 min and 4-fluorobenzeneboronic acid (40.0 mg, 0.290 mmol) was added. The flask was sealed and heated at 100 ^o^C overnight. The reaction mixture was filtered through Celite and the filtrate was concentrated under reduced pressure. The residue was purified by automated chromatography on SiO_2_ (4 g column, stepwise gradient, hexanes to EtOAc over 15 min) to yield a mixture of 1-(*t*-butyl)-6-chloro-3-(4-fluorophenyl)-1*H*-pyrazolo[3,4-*d*]pyrimidin-4-amine and starting material (10:1, 54.0 mg) as an off-white solid.

A solution of this mixture (18.0 mg, 0.0512 mmol), 10% Pd/C (0.08 g, 0.07 mmol) and NH_4_HCO_2_ (20.0 mg, 0.340 mmol) in MeOH (2 mL) was stirred at rt for 15 min, purged with H_2_ for 15 min and stirred under H_2_ overnight. The reaction mixture was filtered through Celite and the filtrate was concentrated under reduced pressure. The residue was purified by chromatography on SiO_2_ (hexanes:EtOAc, 6:1) to yield **1d** (4.10 mg (29%, 93% purity by LC-MS) as a colorless solid: ^1^H NMR (CDCl_3_, 400 MHz) δ 8.36 (s, 1 H), 7.68 (dd, *J* = 8.2, 5.2 Hz, 2 H), 7.23 (t, *J* = 8.2 Hz, 2 H), 5.37 (bs, 2 H), 1.83 (s, 9 H); ^13^C NMR (CDCl_3_, 100 MHz) δ 163.1 (d, *J_CF_* = 247 Hz), 157.7, 154.6, 154.3, 141.0, 130.4 (d, *J_CF_* = 8.3 Hz), 129.9 (d, *J_CF_* = 3.1 Hz), 129.2, 128.6, 116.3 (d, *J_CF_* = 22 Hz), 99.7, 60.5, 29.2; HRMS (ESI) *m/z* calcd for C_15_H_16_FN_5_ (M+H)^+^ 286.1468, found 286.1452.

**3-([1,1'-Biphenyl]-4-yl)-1-(*tert*-butyl)-1*H*-pyrazolo[3,4-*d*]pyrimidin-4-amine (1e).**^1,2,3^ To a solution of dichloro[1,1'-bis(diphenylphosphino)ferrocene]palladium (II)-dichloromethane adduct (18.2 mg, 0.0200 mmol) in freshly distilled and degassed 1,4-dioxane (2 mL) was added 3-bromo-1-(*t*-butyl)-6-chloro-1*H*-pyrazolo[3,4-*d*]pyrimidin-4-amine (**6c**, 75.0 mg, 0.250 mmol), and Cs_2_CO_3_ (122 mg, 0.370 mmol). The resulting mixture was stirred for 10 min and 4-phenylbenzeneboronic acid (58.5 mg, 0.300 mmol) was added. The flask was sealed and heated to 100 ^o^C overnight. The reaction mixture was filtered through Celite and concentrated under reduced pressure. The residue was purified by automated chromatography on SiO_2_ (4 g column, stepwise gradient, hexanes to EtOAc over 15 min) to yield a mixture of 3-([1,1'-biphenyl]-4-yl)-1-(*t*-butyl)-6-chloro-1*H*-pyrazolo[3,4-*d*]pyrimidin-4-amine and starting material (9:1, 35.0 mg) as an off-white solid.

A solution of this mixture (20.0 mg, 0.0477 mmol), 10% Pd/C (75.2 mg, 0.0600 mmol), and NH_4_CO_2_ (20.0 mg, 0.320 mmol) in MeOH (2 mL) was stirred at rt for 30 min, purged with H_2_ for 15 min and stirred under H_2_ overnight. The reaction mixture was filtered through Celite and the filtrate was concentrated under reduced pressure. The residue was purified by chromatography on SiO_2_ (hexanes:EtOAc, 4:1) to yield **1e** (8.90 mg, 54%, 94% purity by LC-MS) as a colorless solid: ^1^H NMR (CDCl_3_, 400 MHz) δ 8.38 (s, 1 H), 7.78 (d, *J* = 8.4 Hz, 2 H), 7.75 (d, *J* = 8.4 Hz, 2 H), 7.65 (d, *J* = 7.6 Hz, 2 H), 7.48 (t, *J* = 7.6 Hz, 2 H), 7.39 (t, *J* = 7.6 Hz, 1 H), 5.84 (bs, 3 H), 1.86 (s, 9 H); ^13^C NMR (CDCl_3_, 100 MHz) δ 157.7, 154.5, 154.3, 141.6 (2 C), 140.3, 132.6, 128.9, 128.9, 127.9, 127.7, 127.0, 99.7, 60.5, 29.2; HRMS (ESI) *m/z* calcd for C_21_H_21_N_5_ (M+H)^+^ 344.1875, found 344.1858.

**1-Methyl-3-(naphthalen-1-yl)-1*H*-pyrazolo[3,4-*d*]pyrimidin-4-amine (1f)**.^1,2^ To a solution of dichloro[1,1'-bis(diphenylphosphino)ferrocene]palladium (II)-dichloromethane adduct (9.80 mg, 0.01 mmol) in freshly distilled and degassed 1,4-dioxane was added 3-bromo-6-chloro-1-methyl-1*H*-pyrazolo[3,4-*d*]pyrimidin-4-amine (**6b**, 50.0 mg, 0.190 mmol) and Cs_2_CO_3_ (90.0 mg, 0.0300 mmol). The reaction mixture was stirred for 10 min and 1-naphthylboronic acid (39.3 mg, 0.230 mmol) was added. The solution was continuously degassed by purging with N_2_ over the course of the addition of reagent. The vial was capped and the solution was stirred at 80 ^o^C overnight and at 100 ^o^C for an additional 12 h, and filtered through Celite. The filtrate was concentrated under reduced pressure and the residue was purified by chromatography on SiO_2_ (hexanes:EtOAc, 2:1) to yield a mixture of 6-chloro-1-methyl-3-(naphthalen-1-yl)-1*H*-pyrazolo[3,4-*d*]pyrimidin-4-amine and starting material (6:1, 18.0 mg) as an off-white solid.

A solution of this mixture (4.20 mg, 0.00857 mmol), 5% Pd/C (81.9 mg, 0.0300 mmol) and NH_4_HCO_2_ (8.70 mg, 0.140 mmol) in MeOH (1 mL) was stirred at rt for 10 min, purged with H_2_ for 30 min and stirred under H_2_ overnight. The reaction mixture was filtered through Celite, which was rinsed with CH_2_Cl_2_ (4 mL), and the combined filtrate was concentrated under reduced pressure. The residue was purified by chromatography on SiO_2_ (hexanes:EtOAc, 6:1) to yield **1f** (3.70 mg, 68%, 96% purity by LC-MS) as a colorless solid: Mp 210-212°C; IR (neat) 3465, 3299, 3220, 2937, 1631, 1565, 1513, 1312, 1269 cm^-1^; ^1^H NMR (CDCl_3_, 400 MHz) δ 8.43 (s, 1 H), 8.01-7.92 (m, 3 H), 7.67-7.52 (m, 4 H), 5.11 (bs, 2 H), 4.16 (s, 3 H); ^13^C NMR (CDCl_3_, 100 MHz) δ 157.6, 156.2, 154.3, 142.4, 133.9, 131.5, 130.0, 129.8, 128.6, 128.4, 127.3, 126.6, 125.5, 125.3, 100.3, 34.0; HRMS (ESI) *m/z* calcd for C_16_H_13_N_5_ (M+H)^+^ 276.1249, found 276.1242.

***N*,1-Dimethyl-3-(naphthalen-1-yl)-1*H*-pyrazolo[3,4-*d*]pyrimidin-4-amine** (**1g)**.^1,2^ A solution of Pd_2_(dba)_3_ (17.6 mg 0.0200 mmol) and PA-Ph (8.70 mg, 0.0300 mmol) in degassed 1,4-dioxane (1.7 mL) was stirred at rt for 10 min, treated with 3-bromo-6-chloro-*N*,1-dimethyl-1*H*-pyrazolo[3,4-*d*]pyrimidin-4-amine (**6a**, 75.0 mg, 0.270 mmol), Cs_2_CO_3_ (133 mg, 0.410 mmol) and 1-naphthylboronic acid (56.0 mg, 0.330 mmol) and stirred in a pre-equilibrated oil bath at 100 ^o^C overnight. The reaction mixture was filtered through Celite and the filtrate was concentrated under reduced pressure. The residue was purified by chromatography on SiO_2_ (hexanes:EtOAc, 4:1) to yield a mixture of 6-chloro-*N*,1-dimethyl-3-(naphthalen-1-yl)-1*H*-pyrazolo[3,4-*d*]pyrimidin-4-amine and starting material (10:1, 34.0 mg) as an off white solid.

A solution of this mixture (8.00 mg, 0.0182 mmol), 10% Pd/C (40.0 mg, 0.0400 mmol) and NH_4_HCO_2_ (9.30 mg, 0.150 mmol) in MeOH (1 mL) was stirred at rt for 30 min, purged with H_2_ for 15 min and stirred under H_2_ overnight. The reaction mixture was filtered through Celite and the filtrate was concentrated under reduced pressure. The residue was purified by chromatography on SiO_2_ (hexanes:EtOAc, 6:1) to yield **1g** (3.30 mg, 51%) as a colorless solid: Mp 154.2-155.6 °C; IR (neat) 3438, 3045, 2939, 1599, 1569, 1312, 1269 cm^-1^; ^1^H NMR (CDCl_3_, 400 MHz) δ 8.51 (s, 1 H), 7.99 (dd, *J* = 16, 8.0 Hz, 2 H), 7.90 (d, *J* = 8.0 Hz, 2 H), 7.70-7.50 (m, 4 H), 4.76 (bs, 1 H), 4.15 (s, 3 H), 2.89 (d, *J* = 4.8 Hz, 3 H); ^13^C NMR (CDCl_3_, 100 MHz) δ 157.5, 156.3, 153.7, 141.8, 134.0, 131.6, 130.3, 129.8, 128.5, 128.3, 127.2, 126.6, 125.5, 125.4, 100.5, 34.0, 27.7; HRMS (ESI) *m/z* calcd for C_17_H_14_ClN_5_ (M+H)^+^ 290.1406, found 290.1385.

**3-(4-Methoxyphenyl)-1-methyl-1*H*-pyrazolo[3,4-*d*]pyrimidin-4-amine (1h)**.^2^ A mixture of 6-chloro-3-(4-methoxyphenyl)-1-methyl-1*H*-pyrazolo[3,4-*d*]pyrimidin-4-amine (**6b**, 13.2 mg, 0.0500 mmol), 5% Pd/C (162 mg, 0.0700 mmol) and NH_4_HCO_2_ (17.2 mg, 0.270 mmol) in MeOH (3 mL) was stirred at rt for 10 min, purged with H_2_ for 10 min and stirred under H_2_ overnight. The reaction mixture was filtered through Celite, which was washed with CH_2_Cl_2_ (4 mL), and the combined filtrate was concentrated under reduced pressure. The residue was purified by chromatography on SiO_2_ (hexanes:EtOAc, 6:1) to yield **1h** (4.40 mg, 37%, 100% purity by LC-MS) as a colorless solid: Mp 185-188 °C IR (neat) 3468, 3071, 2926, 2851, 1647, 1581, 1568, 1322, 1288, 1249, 1172 cm^-1^; ^1^H NMR (CDCl_3_, 400 MHz) δ 8.40 (s, 1 H), 7.62 (d, *J* = 8.8 Hz, 2 H), 7.06 (d, *J* = 8.8 Hz, 2 H), 5.48 (bs, 2 H), 4.07 (s, 3 H), 3.88 (s, 3 H); ^13^C NMR (CDCl_3_, 100 MHz) δ 160.3, 157.8, 156.0, 154.5, 144.0, 129.7, 125.5, 114.8, 98.4, 55.4, 33.8; HRMS (ESI) *m/z* calcd for C_13_H_13_N_5_O (M+H)^+^ 256.1198, found 256.1181.

**3-(4-Methoxyphenyl)-*N*,1-dimethyl-1*H*-pyrazolo[3,4-*d*]pyrimidin-4-amine (1i)**.^2^ A mixture of 6-chloro-3-(4-methoxyphenyl)-*N*-methyl-1*H*-pyrazolo[3,4-d]pyrimidin-4-amine (**6a**, 8.00 mg, 0.03 mmol), 10% Pd/C (0.05 g, 0.10 mmol) and NH_4_HCO_2_ (1.00 mg, 0.160 mmol) in MeOH (0.8 mL) was stirred at rt for 30 min, purged with H_2_ for 15 min and stirred under H_2_ overnight. The reaction mixture was filtered through Celite and the filtrate was concentrated under reduced pressure. The residue was dissolved in EtOAc (1 mL), washed with H_2_O (2 x 1 mL), dried (MgSO_4_) and concentrated under reduced pressure. The crude product was purified by chromatography on SiO_2_ (EtOAc:hexanes, 2:1) to yield **1i** (5.30 mg, 74%) as a colorless solid: Mp 110.2-111.6 °C; IR (neat) 3442, 2935, 1591, 1567,1526, 1496, 1246 cm^-1^; ^1^H NMR (CDCl_3_, 400 MHz) δ 8.46 (s, 1 H) 7.56 (d, *J* = 8.4 Hz, 2 H), 7.06 (d, *J* = 8.4 Hz, 2 H), 5.37 (bs, 1 H), 4.05 (s, 3 H), 3.89 (s, 3 H), 3.09 (d, *J* = 4.8 Hz, 3 H); ^13^C NMR (CDCl_3_, 100 MHz) δ 160.2, 157.8, 156.0, 153.9, 143.5, 129.6, 125.8, 114.8, 98.6, 55.4, 33.7, 27.8; HRMS (ESI) *m/z* calcd for C_14_H_14_ClN_5_O (M+H)^+^ 270.1355, found 270.1351.

**6-Chloro-3-(4-methoxyphenyl)-1-methyl-1*H*-pyrazolo[3,4-*d*]pyrimidin-4-amine (1j)**.^1^ A solution of dichloro[1,1'-bis(diphenylphosphino)ferrocene]palladium (II)-dichloromethane adduct (9.90 mg, 0.0100 mmol) in freshly distilled and degassed 1,4-dioxane (2 mL) was stirred for 10 min, treated with 3-bromo-6-chloro-1-methyl-1*H*-pyrazolo[3,4-*d*]pyrimidin-4-amine (**6b**, 50.0 mg, 0.190 mmol), Cs_2_CO_3_ (90.0 mg, 0.290 mmol), and 4-methoxybenzeneboronic acid (35.0 mg, 0.230 mmol) and stirred at 80 ^o^C overnight and at 100 ^o^C for an additional 4 h. The reaction mixture was filtered through Celite and the filtrate was concentrated under reduced pressure. The residue was purified by chromatography on SiO_2_ (hexanes:EtOAc, 1:1) to yield **1j** (14.0 mg, 25%) as a colorless solid: Mp >250 °C; IR (neat) 3465, 3288, 3070, 3001, 2835, 1648, 1586, 1504, 1450, 1251, 1038 cm^-1^; ^1^H NMR (CDCl_3_, 400 MHz) δ 7.59 (d, *J* = 8.4 Hz, 2 H), 7.06 (d, *J* = 8.4 Hz, 2 H), 5.72 (bs, 2 H), 4.03 (s, 3 H), 3.88 (s, 3 H); ^13^C NMR (CDCl_3_, 100 MHz) δ 160.5, 158.2, 157.9, 155.6, 144.4, 129.5, 124.9, 114.9, 97.1, 55.4, 34.1; HRMS (ESI) *m/z* calcd for C_13_H_12_ClN_5_O (M+H)^+^ 290.0809, found 290.0799.

**6-Chloro-3-(4-methoxyphenyl)-*N*,1-dimethyl-1*H*-pyrazolo[3,4-*d*]pyrimidin-4-amine** (**1k**).^4^ A solution of Pd_2_(dba)_3_ (17.6 mg 0.0200 mmol) and PA-Ph (7.90 mg, 0.0300 mmol) in degassed 1,4-dioxane (1 mL) was stirred for 10 min, treated with 3-bromo-6-chloro-*N*,1-dimethyl-1*H*-pyrazolo[3,4-*d*]pyrimidin-4-amine (**6a**, 74.8 mg, 0.270 mmol), Cs_2_CO_3_ (133 mg, 0.410 mmol) and 4-methoxybenzeneboronic acid (39.0 mg 0.270 mmol), and stirred overnight in a pre-equilibrated oil bath at 50 ^o^C. The reaction mixture was filtered through Celite and the filtrate was concentrated under reduced pressure. The residue was purified by preparative TLC on SiO_2_ (EtOAc:hexanes, 7:3) to yield **1k** (28.4 mg, 35%, 100% purity by LC-MS) as a colorless solid: Mp 159.3-160.9 °C; IR (neat) 3349, 2957, 1601, 1569, 1496, 1246, 1209, 1172 cm^-1^; ^1^H NMR (CDCl_3_, 400 MHz) δ 7.54 (d, *J* = 8.8 Hz, 2 H), 7.06 (d, *J* = 8.8 Hz, 2 H), 5.51 (bs, 1 H), 4.01 (s, 3 H), 3.89 (s, 3 H), 3.09 (d, *J* = 4.8 Hz, 3 H); ^13^C NMR (CDCl_3_, 100 MHz) δ 160.3, 158.2, 157.9, 154.8, 143.6, 129.4, 125.1, 114.8, 97.2, 55.3, 33.8, 27.9; HRMS (ESI) *m/z* calcd for C_14_H_14_ClN_5_O (M+H)^+^ 304.0965, found 304.0974.

***N*,1-Dimethyl-3-(4-(trifluoromethoxy)phenyl)-1*H*-pyrazolo[3,4-*d*]pyrimidin-4-amine (1l)**.^2^ A mixture of 6-chloro-*N*,1-dimethyl-3-(4-(trifluoromethoxy)phenyl)-1*H*-pyrazolo[3,4-*d*]pyrimidin-4-amine (**6a**, 8.10 mg, 0.0200 mmol), 5% Pd/C (80.0 mg, 0.0300 mmol) and NH_4_HCO_2_ (8.50 mg, 0.140 mmol) in MeOH (2 mL) was stirred at rt for 30 min, purged with H_2_ for 15 min and stirred under H_2_ overnight. The reaction mixture was filtered through Celite and the filtrate was concentrated under reduced pressure. The residue was dissolved in EtOAc (1 mL), washed with H_2_O (2 x 1 mL), dried (MgSO_4_) and concentrated under reduced pressure. The crude product was purified by chromatography on SiO_2_ (hexanes:EtOAc, 6:1) to yield **1l** (6.50 mg, 89%, 91% purity by LC-MS) as a colorless solid: Mp 101.5-103.3 °C; IR (neat) 3452, 2943, 1592, 1568, 1249, 1219, 1204, 1161, 1103 cm^-1^; ^1^H NMR (CDCl_3_, 400 MHz) δ 8.49 (s, 1 H), 7.69 (d, *J* = 8.4 Hz, 2 H), 7.39 (d, *J* = 8.4 Hz, 2 H), 5.25 (bd, *J* = 2.4 Hz, 1 H), 4.10 (s, 3 H), 3.12 (d, *J* = 5.2 Hz, 3 H); ^13^C NMR (CDCl_3_, 100 MHz) δ 157.6, 156.1, 154.1, 149.7, 142.1, 132.6, 129.9, 129.4, 128.4, 121.8, 120.4 (q, *J_CF_* = 257 Hz), 98.6, 33.9, 28.0; HRMS (ESI) *m/z* calcd for C_14_H_11_ClF_3_N_5_O (M-H)^-^ 324.1072, found 324.1078.

**6-Chloro-*N*,1-dimethyl-3-(4-(trifluoromethoxy)phenyl)-1*H*-pyrazolo[3,4-*d*]pyrimidin-4-amine** **(1m)**.^1^ A solution of Pd_2_(dba)_3_ (17.6 mg, 0.0200 mmol) and dppf (9.00 mg, 0.0160 mmol) in freshly distilled and degassed 1,4-dioxane (1.7 mL) was stirred for 10 min, treated with 3-bromo-6-chloro-*N*,1-dimethyl-1*H*-pyrazolo[3,4-*d*]pyrimidin-4-amine (**6a**, 75.0 mg, 0.270 mmol), Cs_2_CO_3_ (133 mg, 0.410 mmol), and 4-trifluoromethoxybenzeneboronic acid (67.0 mg, 0.330 mmol), and stirred in a pre-heated oil at 100 ^o^C for 2 d. The reaction mixture was filtered through Celite and the filtrate was concentrated under reduced pressure. The residue was purified by chromatography on SiO_2_ (hexanes:EtOAc, 4:1) to yield **1m** (27.0 mg, 28%, 100% purity by LC-MS) as a colorless solid: Mp 115.3-119.1 °C; IR (neat) 3444, 2944, 1593, 1567, 1246, 1202, 1155 cm^-1^; ^1^H NMR (CDCl_3_, 400 MHz) δ 7.66 (d, *J* = 8.4 Hz, 2 H), 7.38 (d, *J* = 8.4 Hz, 2 H), 5.40 (bs, 1 H), 4.00 (s, 3 H), 3.11 (d, *J* = 4.8 Hz, 3 H); ^13^C NMR (CDCl_3_, 100 MHz) δ 158.5, 157.9, 155.2, 149.9, 142.4, 131.7, 129.8, 121.7, 120.4 (q, *J_CF_* = 257 Hz), 97.3, 34.1, 28.2; HRMS (ESI) *m/z* calcd for C_14_H_11_ClF_3_N_5_O (M-H)^-^ 356.0526, found 356.0521.

**3-(4-Fluorophenyl)-*N*,1-dimethyl-1H-pyrazolo[3,4-*d*]pyrimidin-4-amine** (**1n**).^2^ A solution of 6-chloro-3-(4-fluorophenyl)-*N*-methyl-1*H*-pyrazolo[3,4-*d*]pyrimidin-4-amine (**6a**, 12.0 mg, 0.0400 mmol), 10% Pd/C (58.0 mg, 0.0490 mmol) and NH_4_HCO_2_ (5.30 mg, 0.0800 mmol) in MeOH (1 mL) was stirred at rt for 30 min, purged with H_2_ for 15 min and stirred under H_2_ overnight. The reaction mixture was filtered through Celite and the filtrate was concentrated under reduced pressure. The residue was dissolved in EtOAc (1 mL), washed with H_2_O (2 x 1 mL), dried (MgSO_4_) and concentrated under reduced pressure. The residue was purified by chromatography on SiO_2_ (hexanes:EtOAc, 1:1 to 1:4) to yield **1n** (8.00 mg, 76%, 96% purity by LC-MS) as a colorless solid: Mp 111.8-114.5 °C; IR (neat) 3446, 2941, 1588, 1567, 1522, 1494, 1312, 1265, 1220 cm^-1^; ^1^H NMR (CDCl_3_, 400 MHz) δ 8.47 (s, 1 H), 7.62 (dd, *J* = 8.4, 5.2 Hz, 2 H), 7.23 (t, *J* = 8.4 Hz, 2 H), 5.26 (bs, 1 H), 4.06 (s, 3 H), 3.10 (d, *J* = 4.8 Hz, 3 H); ^13^C NMR (CDCl_3_, 100 MHz) δ 163.2 (d, *J_CF_* = 248 Hz), 157.6, 156.1, 154.0, 142.6, 130.2 (d, *J_CF_* = 82 Hz), 129.2 (d, *J_CF_* = 3.2 Hz), 116.4 (d, *J_CF_* = 22 Hz), 98.6, 33.8, 27.9; HRMS (ESI) *m/z* calcd for C_13_H_12_FN_5_ (M+H)^+^ 258.1155, found 258.1150.

**6-Chloro-3-(4-fluorophenyl)-*N*,1-dimethyl-1*H*-pyrazolo[3,4-*d*]pyrimidin-4-amine** (**1o**).^4^ A solution of Pd_2_(dba)_3_ (17.6 mg 0.0200 mmol) and PA-Ph (7.90 mg, 0.0300 mmol) in 1,4-dioxane (1 mL) was stirred for 10 min, treated with 3-bromo-6-chloro-*N*,1-dimethyl-1*H*-pyrazolo[3,4-*d*]pyrimidin-4-amine (**6a**, 74.8 mg, 0.270 mmol), Cs_2_CO_3_ (133 mg, 0.410 mmol) and 4-fluorobenzeneboronic acid (42.0 mg 0.300 mmol), and stirred at 60 ^o^C overnight and at 100 ^o^C for an additional 4 h. The reaction mixture was filtered through Celite and the filtrate was concentrated under reduced pressure. The residue was purified by chromatography on SiO_2_ (hexanes:EtOAc, 5:1) to yield **1o** (21.0 mg, 27%, 100% purity by LC-MS) as a colorless solid: Mp 189.9-192.3 °C; IR (neat) 3438, 2943, 1597, 1569, 1526, 1493, 1347, 1260 cm^-1^; ^1^H NMR (CDCl_3_, 400 MHz) δ 7.60 (dd, *J* = 8.4, 5.6 Hz, 2 H), 7.20-7.30 (m, 2 H), 5.40 (bs, 1 H), 4.02 (s, 3 H), 3.11 (d, *J* = 4.8 Hz, 3 H); ^13^C NMR (CDCl_3_, 100 MHz) δ 163.4 (d, *J_CF_* = 248 Hz), 158.5, 158.0, 155.1, 142.9, 130.2 (d, *J_CF_* = 82 Hz), 129.2 (d, *J_CF_* = 3.4 Hz), 116.7 (d, *J_CF_* = 21.4 Hz), 97.4, 34.1, 28.2; HRMS (ESI) *m/z* calcd for C_13_H_11_ClFN_5_ (M+H)^+^ 292.0765, found 292.0788.

**3-([1,1'-Biphenyl]-4-yl)-*N*,1-dimethyl-1*H*-pyrazolo[3,4-*d*]pyrimidin-4-amine (1p)**.^2^ A solution of 3-([1,1'-biphenyl]-4-yl)-6-chloro-*N*,1-dimethyl-1*H*-pyrazolo[3,4-*d*]pyrimidin-4-amine (**6a**, 20.0 mg, 0.0500 mmol), 10% Pd/C (85.0 mg, 0.0725 mmol) and NH_4_HCO_2_ (18.3 mg, 0.290 mmol) in MeOH (2 mL) was stirred at rt for 30 min, purged with H_2_ for 30 min and stirred under H_2_ overnight. The reaction mixture was filtered through Celite and the filtrate was concentrated under reduced pressure. The residue was purified by chromatography on SiO_2_ (hexanes:EtOAc, 6:1) to yield **1p** (12.8 mg, 84%, 100% purity by LC-MS) as a colorless solid: Mp 190.8-192.3 °C; IR (neat) 3439, 3027, 2937, 1590, 1566, 1487, 1264 cm^-1^; ^1^H NMR (CDCl_3_, 400 MHz) δ 8.49 (s, 1 H), 7.78-7.71 (m, 4 H), 7.66 (d, *J* = 7.6 Hz, 2 H), 7.49 (t, *J* = 7.6 Hz, 2 H), 7.40 (t, *J* = 7.2 Hz, 1 H), 5.46 (bd, *J* = 4.0 Hz, 1 H), 4.08 (s, 3 H), 3.12 (d, *J* = 4.8 Hz, 3 H); ^13^C NMR (CDCl_3_, 100 MHz) δ 157.7, 156.0, 154.0, 143.3, 141.8, 140.1, 132.4, 128.9, 128.7, 128.0, 127.8, 127.1, 98.7, 33.8, 27.9; HRMS (ESI) *m/z* calcd for C_6_H_6_ClN_5_ (M+H)^+^ 316.1562, found 316.1571.

**3-([1,1'-Biphenyl]-4-yl)-6-chloro-*N*,1-dimethyl-1*H*-pyrazolo[3,4-*d*]pyrimidin-4-amine (1q)**.^1^ A solution of Pd_2_(dba)_3_ (17.6 mg, 0.02 mmol) and dppf (9.80 mg, 0.0160 mmol) in distilled and degassed 1,4-dioxane (1.7 mL) was stirred for 10 min, treated with 3-bromo-6-chloro-*N*,1-dimethyl-1*H*-pyrazolo[3,4-*d*]pyrimidin-4-amine (**6a**, 75.0 mg, 0.270 mmol), Cs_2_CO_3_ (133 mg, 0.41 mmol) and 4-phenylbenzeneboronic acid (64.0 mg, 0.330 mmol), and stirred in a pre-heated oil bath at 100 ^o^C overnight. The reaction mixture was filtered through Celite and the filtrate was concentrated under reduced pressure. The residue was purified by chromatography on SiO_2_ (hexanes:EtOAc, 4:1) to yield **1q** (47.0 mg, 50%) as a colorless solid: Mp 211.9-213.8 °C; IR (neat) 3435, 3058, 1593, 1569, 1485, 1347, 1260 cm^-1^; ^1^H NMR (CDCl_3_, 400 MHz) δ 7.77-7.66 (m, 4 H), 7.65 (d, *J* = 7.6 Hz, 2 H), 7.48 (t, *J* = 7.2 Hz, 2 H), 7.40 (t, *J* = 7.2 Hz, 1 H), 5.61 (bs, 1 H), 4.02 (s, 3 H), 3.11 (d, *J* = 6.0 Hz, 3 H); ^13^C NMR (CDCl_3_, 100 MHz) δ 158.4, 158.0, 155.1, 143.6, 142.0, 139.9, 131.8, 128.9, 128.6, 128.1, 127.8, 127.0, 97.3, 34.0, 28.1; HRMS (ESI) *m/z* calcd for C_19_H_16_ClN_5_ (M-H)^-^ 350.1172, found 350.1165.

**2,4,6-Trichloropyrimidine-5-carbaldehyde** (**3**).^5^ *N*,*N*-Dimethylformamide (4.85 mL, 62.5 mmol) was added dropwise to phosphorus oxychloride (25.1 mL, 269 mmol) at 0 ºC. Barbituric acid (5.00 g, 62.5 mmol) was then added portion-wise over 1 h, and the resulting suspension was stirred in a pre-equilibrated oil bath at 90 ºC for 2 d. The reaction mixture was concentrated under reduced pressure and a water-ice mixture (50 mL) was slowly added to the residue in an ice bath. The yellow precipitate that formed was filtered, washed with a water-ice mixture (3 x 10 mL), dissolved in CH_2_Cl_2_ (100 mL) and washed with brine (30 mL) and sat. aqueous NaHCO_3_ (30 mL). The organic extract was dried (MgSO_4_) and concentrated under reduced pressure to yield **3** (4.70 g, 57%) as a pale yellow solid: ^1^H NMR (CDCl_3_, 400 MHz) δ 10.40 (s, 1 H); ^13^C NMR (CDCl_3_, 100 MHz) δ 184.6, 164.0, 161.5, 160.9, 159.9, 122.9.

**4,6-Dichloro-1-methyl-1*H*-pyrazolo[3,4-*d*]pyrimidine** (**4a**).^5^ To a solution of **3** (1.00 g, 4.73 mmol) in dry THF (10 mL) at -78 ºC was added dropwise methyl hydrazine (0.26 mL, 4.73 mmol) in dry THF (2 mL) followed by Et_3_N (2.0 mL, 14.2 mmol). The resulting reaction mixture was stirred for 1.5 h at -78 ºC. The solvent was removed under reduced pressure and the crude product was purified by chromatography on SiO_2_ (hexanes:EtOAc, 8:2) to yield **4a** (0.570 g, 59%) as a colorless solid: Mp 88.9-90.9 °C; IR (neat) 3110, 1588, 1545, 1371, 1299, 1127 cm^-1^; ^1^H NMR (CDCl_3_, 400 MHz) δ 8.12 (s, 1 H), 4.10 (s, 3 H); ^13^C NMR (CDCl_3_, 100 MHz) δ 156.5, 155.4, 154.2, 132.3, 112.6, 34.6.

**1-(*t*-Butyl)-4,6-dichloro-1*H*-pyrazolo[3,4-*d*]pyrimidine (4b)**.^5^ To a solution of **3** (1.00 g, 4.73 mmol) in dry CH_2_Cl_2_ (20 mL) at -78 ºC under Ar was added dropwise a solution of *t*-butyl hydrazine hydrochloride (631 mg, 4.73 mmol) and Et_3_N (1.435 g, 2.0 mL, 14.2 mmol) in dry CH_3_CN (30 mL). The reaction mixture was stirred for 2 h at -78 ^o^C. The solvent was removed was under reduced pressure and the residue was purified by chromatography on SiO_2_ (hexanes:EtOAc, 8:1) to yield **4b** (937 mg, 81%) as a colorless solid: IR (neat) 3118, 2984, 2934, 1582, 1525, 1320, 1230, 1208, 1152 cm^-1^; ^1^H NMR (CDCl_3_, 400 MHz) δ 8.08 (s, 1 H), 1.80 (s, 9 H); ^13^C NMR (CDCl_3_, 100 MHz) δ 155.1, 155.0, 153.6, 130.8, 113.7, 61.9, 29.0; HRMS (ESI) *m/z* calcd for C_9_H_10_C_l2_N_4_ (M+H)^+^ 245.0361, found 245.0357.

**6-Chloro-*N*,1-dimethyl-1*H*-pyrazolo[3,4-*d*]pyrimidin-4-amine** (**5a**). ^6^ To a solution of **4a** (300 mg, 1.48 mmol) in dry THF (15 mL) was added methylamine hydrochloride (0.110 g, 1.55 mmol), followed by freshly distilled Et_3_N (1.04 mL, 7.38 mmol). The reaction mixture was stirred at rt for 2 d, concentrated under reduced pressure, and the residue was dissolved in EtOAc (100 mL), washed with H_2_O (3 x 75 mL), dried (MgSO_4_) and concentrated under reduced pressure. The crude product was purified by chromatography on SiO_2_ (hexanes:EtOAc, 2:1) to yield **5a** (267 mg, 91%) as a colorless solid: Mp 239.3-240.4 °C; IR (neat) 3230, 3105, 1614, 1579, 1316, 1128, 930 cm^-1^; ^1^H NMR (DMSO-d_6_, 400 MHz) δ 8.70 (bs, 1 H), 8.03 (s, 1 H), 3.83 (s, 3 H), 2.95 (d, *J* = 4.4 Hz, 3 H); ^13^C NMR (DMSO-d_6_, 100 MHz) δ 157.4, 157.2, 153.3, 131.6, 99.3, 33.7, 27.1; HRMS (ESI) *m/z* calcd for C_7_H_8_ClN_5_ (M+H)^+^ 198.0546, found 198.0551.

**6-Chloro-1-methyl-1*H*-pyrazolo[3,4-*d*]pyrimidin-4-amine** (**5b**).^6^ Ammonia gas was bubbled for 15 min through a suspension of **4a** (0.30 g, 1.48 mmol) in dry THF (48 mL). The mixture was stirred at rt for 8 h, concentrated under reduced pressure, and the residue was dissolved in EtOAc (30 mL), washed with H_2_O (3 x 15 mL), dried (MgSO_4_) and concentrated under reduced pressure. The crude product was purified by chromatography on SiO_2_ (hexanes:EtOAc, 1:1 to 100% EtOAc) to yield **5b** (220 mg, 81%) as a colorless solid: Mp 265-275 °C; IR (neat) 3308, 3099, 2920, 1657, 1599, 1561, 1187 cm^-1^; ^1^H NMR (DMSO-d_6_, 400 MHz) δ 8.24 (bs, 1 H), 8.11 (bs, 1 H), 8.05 (s, 1 H), 3.83 (s, 3 H); ^13^C NMR (DMSO-d_6_, 100 MHz) δ 158.6, 157.3, 153.9, 132.1, 98.9, 33.6; HRMS (ESI) *m/z* calcd for C_6_H_6_ClN_5_ (M+H)^+^ 184.0390, found 184.0381.

**1-(*t*-Butyl)-6-chloro-1*H*-pyrazolo[3,4-*d*]pyrimidin-4-amine (5c)**.^5^ Ammonia gas was bubbled through a suspension of **4b** (800 mg, 3.26 mmol) in THF (100 mL) for 20 min. A white precipitate started to form after several minutes and the resulting mixture was stirred at rt overnight. The solvent was removed under reduced pressure and the residue was dissolved in EtOAc (250 mL), washed with H_2_O (3 x 75 mL), dried (Mg_2_SO_4_) and concentrated under reduced pressure to yield **5c** (0.740 g, 98%) as a colorless solid which was used in the next step without further purification: Mp 272-274 °C; IR (neat) 3297, 3135, 2971, 2932, 1644, 1592, 1469, 1366, 1236, 1171 cm^-1^; ^1^H NMR (DMSO-d_6_, 400 MHz) δ 8.17 (bs, 1 H), 8.03 (s, 2 H), 1.65 (s, 9 H); ^13^C NMR (DMSO-d_6_, 100 MHz) δ 158.7, 156.0, 153.5, 130.6, 100.3, 59.6, 28.7; HRMS (ESI) *m/z* calcd for C_9_H_12_ClN_5_ (M+H)^+^ 226.0859, found 226.0882.

**1-(*t*-Butyl)-6-chloro-*N*-methyl-1*H*-pyrazolo[3,4-*d*]pyrimidin-4-amine (5d)**.^5^ To a solution of **4b** (0.800 g, 3.26 mmol) in THF (24 mL) under Ar was added at rt methylamine hydrochloride (0.230 g, 3.43 mmol) and Et_3_N (2.30 mL, 16.3 mmol). The reaction mixture was stirred at rt overnight, concentrated under reduced pressure, dissolved in EtOAc (75 mL), washed with H_2_O (3 x 30 mL), dried (MgSO_4_) and concentrated under reduced pressure. The residue was purified by chromatography on SiO_2_ (hexanes:EtOAc, 2:1) to yield **5d** (700 mg, 90%) as a colorless solid: Mp 160.7-161.9°C; IR (neat) 3236, 3133, 2947, 1579, 1428, 1361, 1310, 1282, 1124, 1075 cm^-1^; ^1^H NMR (DMSO-d_6_, 400 MHz) δ 8.63 (bd, *J* = 4.4 Hz, 1 H), 8.02 (s, 1 H), 2.93 (d, *J* = 4.4 Hz, 3 H), 1.65 (s, 9 H); ^13^C NMR (DMSO-d_6_, 100 MHz) δ 157.3, 156.1, 152.8, 130.0, 100.7, 59.7, 28.7, 27.0; HRMS (ESI) *m/z* calcd for C_10_H_14_ClN_5_ (M+H)^+^ 240.1016, found 240.1006.

**__**

**3-Bromo-6-chloro-*N*,1-dimethyl-1*H*-pyrazolo[3,4-*d*]pyrimidin-4-amine (6a)**.^4^ A mixture of **5a** (0.260 g, 1.31 mmol) and Br_2_ (0.21 mL, 3.92 mmol) in H_2_O (90 mL) was stirred at rt for 1 h, heated at 100 °C for 2 h, extracted with EtOAc (3 x 75 ml), washed with 5% aqueous NaHSO_3_ solution (100 mL) and brine (75 mL), dried (MgSO_4_) and concentrated under reduced pressure to yield **6a** (318 mg, 88%) as a colorless solid, which was used in the next step without further purification: Mp 246.9-248.0 °C; IR (neat) 3407, 2939, 1606, 1567, 1325, 1200 cm^-1^; ^1^H NMR (CDCl_3_, 400 MHz) δ 6.14 (bs, 1 H), 3.94 (s, 3 H), 3.20 (d, *J* = 4.8 Hz, 3 H); ^13^C NMR δ 159.3, 157.4, 154.8, 116.8, 99.3, 34.4, 28.1; HRMS (ESI) *m/z* calcd for C_7_H_7_BrClN_5_ (M+H)^+^ 275.9652, found 275.9652.

**3-Bromo-6-chloro-1-methyl-1*H*-pyrazolo[3,4-*d*]pyrimidin-4-amine** (**6b**).^4^ A mixture **of** **5b** (196 mg, 1.07 mmol) and Br_2_ (0.230 mL, .4.27 mmol) in H_2_O (5 mL) was stirred at rt for 1 h and at 100 °C for an additional 1 h, extracted with EtOAc (3 x 15 mL), and washed with 5% aqueous NaHSO_3_ solution (10 mL) and brine (15 mL). The organic layer was dried (MgSO_4_) and concentrated under reduced pressure. The residue was purified by chromatography on SiO_2_ (hexanes:EtOAc, 1:1) to yield **6b** (113 mg, 40%) as a colorless solid: Mp 285-295 °C; IR (neat) 3451, 3282, 3125, 1586, 1552, 1248, 1189 cm^-1^; ^1^H NMR (DMSO-d_6_, 400 MHz) δ 8.53 (bs, 1 H), 7.28 (bs, 1 H), 3.81 (s, 3 H); ^13^C NMR (DMSO-d_6_, 100 MHz) δ 157.9 (2 C), 154.8, 117.3, 98.5, 34.0; HRMS (ESI) *m/z* calcd for C_6_H_6_ClN_5_ (M+H)^+^ 262.9573, found 262.9593.

**3-Bromo-1-(*t*-butyl)-6-chloro-1*H*-pyrazolo[3,4-*d*]pyrimidin-4-amine (6c)**.^4^ A mixture of **5c** (100 mg, 0.440 mmol) and Br_2_ (0.0500 mL, 0.890 mmol) in H_2_O (5 mL) was stirred at rt for 1 h and then at 100 °C for 30 min. In order to increase the solubilizing power of the reaction mixture, EtOAc (3 mL) was added, and the resulting solution was heated at reflux for an additional 15 min. The two layers were separated and the aqueous phase was extracted with EtOAc (3 x 7.5 mL). The combined organic extracts were washed with 5% aqueous NaHSO_3_ solution (10 mL), brine (15 mL), dried (MgSO_4_) and concentrated under reduced pressure. The residue was purified by chromatography on SiO_2_ (CH_2_Cl_2_ to CH_2_Cl_2_:EtOAc, 1:1) to yield **6c** (110 mg, 84%) as a light yellow solid: Mp 225-226°C; IR (neat) 3450, 3282, 2971, 2919, 2718, 1651, 1634, 1584, 1543, 1461, 1234, 1174, 1131 cm^-1^; ^1^H NMR (CDCl_3_, 400 MHz) δ 6.05 (bs, 2 H), 1.74 (s, 9 H); ^13^C NMR (CDCl_3_, 100 MHz) δ 157.6, 157.1, 154.7, 115.8, 100.1, 61.9, 29.1; HRMS (ESI+) *m/z* calcd for C_9_H_11_BrClN_5_ (M+H)^+^ 303.9965, found 303.9983.

**3-Bromo-1-(*t*-butyl)-6-chloro-*N*-methyl-1*H*-pyrazolo[3,4-*d*]pyrimidin-4-amine (6d)**.^4^ A mixture **of** **5d** (0.120 g, 0.500 mmol) and Br_2_ (0.0500 mL, 1.00 mmol) in H_2_O (3 mL) was stirred at rt for 1 h, treated with EtOAc (1 mL), and heated at reflux for an additional 1 h. Additional EtOAc (3 mL) was added and the reaction mixture was heated at reflux for 15 min. After the starting material had been consumed (TLC, hexanes:EtOAc, 2:1), the layers were separated and the aqueous phase was extracted with EtOAc (3 x 8 mL). The combined organic extracts were washed with 5% aqueous NaHSO_3_ solution (10 mL), brine (15 mL), dried (MgSO_4_) and concentrated under reduced pressure. The residue was purified by automated chromatography on SiO_2_ (12 g column, stepwise gradient, hexanes to EtOAc over 15 min) to yield **6d** (130 mg, 71%) as a colorless solid: Mp 106.8-108.4 °C; IR (neat) 3413, 2982, 2934, 1609, 1553, 1364, 1322, 1217, 1133 cm^-1^; ^1^H NMR (CDCl_3_, 400 MHz) δ 6.12 (bs, 1 H), 3.18 (d, *J* = 4.8 Hz, 3 H), 1.73 (s, 9 H); ^13^C NMR (CDCl_3_, 100 MHz) δ 157.7, 157.3, 154.2, 114.9, 100.2, 61.7, 29.1, 27.9; HRMS (ESI) *m/z* calcd for C_10_H_13_BrClN_5_ (M+H)^+^ 318.0121, found 318.0110.

**References**

1. Wishart, N.; Calderwood, D.; Ericsson, A. M.; Fiamengo, B. R.; Frank,  K. E.; Friedman, M.; George, D. M.; Goedken, E. R.; Josephsohn, N. S.; Li, B. C.; Morytko, M. J.; Stewart, K. D.; Voss, J. W.; Wallace, G. A.; Wang, L.; Woller, K. R.; Breinlinger,  E. C.; Mullen, K. D.; Somal, G. Novel tricyclic compounds as SYK inhibitors and their preparation and use in the treatment of immune and oncological diseases. US Patent. 0190489 A1, 2011.
2. Betschmann, P.; Carroll, W. A.; Ericsson, A. M.; Fix-Stenzel, S. R.; Friedman, M.; Hirst, G. C.; Josephsohn, N. S.; Li, B.; Perez-Medrano, A.; Morytko, M. J.; Rafferty, P.; Chen, H. Piperazines as P2X7 antagonists. US Patent 0076924 A1, 2008.
3. Bulawa, C. E.; Devit, M.; Elbaum, D. Modulation of protein trafficking. US Patent. 0331297 A1, 2010.
4. Todorovic, N.; Awuah, E.; Shakya, T.; Wright, G.D.; Capretta. A. *Tetrahedron Lett.* **2011**, *52*, 5761.
5. Verheijen, J. C.; Richard, D .J.; Curran, K.; Kaplan, J.; Lefever, M.; Nowak, P.; Malwitz, D. J.; Brooijmans, N.; Toral-Barza, L.; Zhang, W. G. *J. Med. Chem*. **2009**, *52*, 8010.
6. Oumata, N.; Bettayeb, K.; Ferandin, Y.; Demange, L.; Giral, A-L.; Goddard, M.-L.; Myrianthopoulos, V.; Mikros, E.; Flajolet, M.; Greengard, P.; Meijer, L.; Galons. H. *J. Med. Chem.* **2008**, *51*, 5229.
7. Wishart, N.; Calderwood, D.; Ericsson, A. M.; Fiamengo, B. R.; Frank,  K. E.; Friedman, M.; George, D. M.; Goedken, E. R.; Josephsohn, N. S.; Li, B. C.; Morytko, M. J.; Stewart, K. D.; Voss, J. W.; Wallace, G. A.; Wang, L.; Woller, K. R.; Breinlinger,  E. C.; Mullen, K. D.; Somal, G. Novel tricyclic compounds as SYK inhibitors and their preparation and use in the treatment of immune and oncological diseases. US Patent. 0190489 A1, 2011.
8. Betschmann, P.; Carroll, W. A.; Ericsson, A. M.; Fix-Stenzel, S. R.; Friedman, M.; Hirst, G. C.; Josephsohn, N. S.; Li, B.; Perez-Medrano, A.; Morytko, M. J.; Rafferty, P.; Chen, H. Piperazines as P2X7 antagonists. US Patent 0076924 A1, 2008.
9. Bulawa, C. E.; Devit, M.; Elbaum, D. Modulation of protein trafficking. US Patent. 0331297 A1, 2010.
