# Supplementary material for: New Pyrazolopyrimidine Inhibitors of Protein Kinase D as Potent Anticancer Agents for Prostate Cancer Cells
Source: PLoS One. 2013 Sep 23;8(9):e75601. doi: 10.1371/journal.pone.0075601 (PMC3781056; doi:10.1371/journal.pone.0075601)
Supplement: Table S1 — The specificity of 1-NM-PP1 in a kinome scan. The activity of 1-NM-PP1, along with 178 known kinase inhibitors, was profiled against a panel of 300 recombinant human protein kinases at a concentration of 0.5 µM in the presence of 10 µM ATP. Data were extracted at a cut-off of <50% residual kinase activity from the Kinase Inhibitor Resource (KIR) online tool (http://kir.fccc.edu/). The kinase activity was determined using a radiometric HotSpot assay which directly measures kinase catalytic activity toward a specific substrate. (DOCX) [file pone.0075601.s003.docx]

Table S1. The specificity of 1-NM-PP1 in a kinome scan. The activity of 1-NM-PP1, along with 178 known kinase inhibitors, was profiled against a panel of 300 recombinant human protein kinases at a concentration of 0.5 μM in the presence of 10 μM ATP. Data were extracted at a cut-off of < 50% residual kinase activity from the Kinase Inhibitor Resource (KIR) online tool (<http://kir.fccc.edu/>). The kinase activity was determined using a radiometric HotSpot assay which directly measures kinase catalytic activity toward a specific substrate.

| Numerical ID | Kinase | % residual kinase activity |
| --- | --- | --- |
|  |  |  |
| 1 | ACK1 | 2.4 |
| 2 | BMX/ETK | 25.2 |
| 3 | BRK | 19.2 |
| 4 | BTK | 41.7 |
| 5 | CK1epsilon | 1.5 |
| 6 | CSK | 46.1 |
| 7 | EPHA1 | 29.8 |
| 8 | EPHA2 | 42.0 |
| 9 | EPHA4 | 48.8 |
| 0 | EPHA5 | 43.5 |
| 11 | EPHA6 | 9.4 |
| 1 | EPHB1 | 47.7 |
| 2 | EPHB2 | 44.6 |
| 13 | EPHB3 | 45.2 |
| 14 | EPHB4 | 30.6 |
| 15 | FYN | 44.7 |
| 16 | HGK MAP4K4 | 43.5 |
| **17** | PKCmu/PRKD1 | 26.2 |
| **18** | PKCnu/PRKD3 | 20.9 |
| **19** | PKD2/PRKD2 | 30.7 |
| 20 | RET | 25.4 |
| 21 | TXK | 28.6 |
| 22 | YES/YES1 | 18.6 |
